# A Systematic Review and Meta-Analysis of the Efficacy and Safety of Rasagiline or Pramipexole in the Treatment of Early Parkinson's Disease

**DOI:** 10.1155/2024/8448584

**Published:** 2024-01-16

**Authors:** Pauli Seppänen, Markus M. Forsberg, Miia Tiihonen, Heikki Laitinen, Selena Beal, David C. Dorman

**Affiliations:** ^1^University of Eastern Finland, Faculty of Health Sciences, School of Pharmacy, Kuopio, Finland; ^2^University of Eastern Finland Library, Kuopio, Finland; ^3^North Carolina State University, College of Veterinary Medicine, Raleigh, NC, USA

## Abstract

**Background:**

Rasagiline or pramipexole monotherapy has been suggested for the management of early Parkinson's disease (PD). The aim of this research was to systematically review the clinical efficacy and safety of rasagiline or pramipexole in early PD (defined as disease duration ≤5 years and Hoehn and Yahr stage of ≤3).

**Methods:**

Randomized controlled trials (RCTs) of rasagiline or pramipexole for early PD published up to September 2021 were retrieved. Outcomes of interest included changes in the Unified Parkinson's Disease Rating Scale (UPDRS) Parts II and III and the incidence of adverse events. Standardized mean difference (SMD), odds ratio (OR), and 95% confidence interval (CI) were calculated, and heterogeneity was measured with the *I*^2^ test.

**Results:**

Nine rasagiline and eleven pramipexole RCTs were included. One post hoc analysis of one rasagiline study was included. Five studies for each drug were included in meta-analyses of the UPDRS scores. The rasagiline meta-analysis focused on patients receiving 1 mg/day. Rasagiline and pramipexole significantly improved UPDRS Part II and III scores when compared to placebo. Significant heterogeneity among the studies was present (*I*^2^ > 70%). Neither rasagiline nor pramipexole increased the relative risk for any adverse events, serious adverse events, or adverse events leading to withdrawal when compared with placebo.

**Conclusion:**

Applying a Grading of Recommendations, Assessment, Development, and Evaluations (GRADE) approach to summarize the evidence, we found moderate confidence in the body of evidence for the efficacy of rasagiline or pramipexole in early PD, suggesting further well-designed, multicenter comparative RCTs remain needed.

## 1. Introduction

Parkinson's disease (PD) is a progressive neurodegenerative disorder characterized clinically by bradykinesia, tremor, rigidity, and postural instability and histologically by neuronal inclusions composed of *α*-synuclein. Nonmotor symptoms including olfactory dysfunction, rapid eye movement, sleep behavior disorder, mood disorders, and autonomic dysfunction often precede the appearance of motor symptoms by several months or years [1, 2]. These motor and nonmotor symptoms can adversely affect a patient's quality of life [3]. The incidence of PD is rising and increases with age [4, 5].

Early management of PD could prolong the ability of a patient to stay in working life and improve their overall quality of life [6, 7]. Levodopa is widely considered one of the most effective treatments for PD, but its use is often delayed because of drug-induced dyskinesias and wearing-off and on-off fluctuations [8, 9]. Alternatives to levodopa in early pharmacologic treatment of PD are dopamine agonists and monoamine oxidase B (MAO-B) inhibitors [10, 11]. A meta-analysis by Chang and coworkers (2017) [12] showed the irreversible inhibitor of MAO-B inhibitor rasagiline to have benefits both as a monotherapy and in combination with another intervention. The safety profile of rasagiline was similar to that of placebo in a systematic review of its use in PD patients [13]. Pramipexole, a nonergoline, D3-preferring dopamine agonist, is another treatment option for the management of motor symptoms associated with PD [14]. A recent systematic review showed that combined pramipexole and levodopa therapy was superior to levodopa monotherapy for the improvement of clinical symptoms in PD patients [15].

Prior systematic reviews evaluating the efficacy and safety of either rasagiline or pramipexole have evaluated patient populations at all stages of the disease. The goal of this study was to focus on the safety and efficacy of these drugs in early PD. Defining early PD in our review was based on the duration of PD in affected patients (five years or less) as well as the presence of mild to moderate symptoms based on the Hoehn and Yahr scale. The Hoehn and Yahr scale is commonly used in clinical studies to stage the level of functional disability and impairment seen in PD patients [16].

## 2. Review Question

We performed a systematic review of the literature to address the following two questions: (a) Are either pramipexole or rasagiline effective in the treatment of early PD; and (b) are either drug associated with adverse events when used for the treatment of early PD?

## 3. Materials and Methods

The protocol for this systematic review was registered on PROSPERO (ID: CRD42021223686). This systematic review did not involve human nor animal data collection. Therefore, ethical approval was not required. The data extraction and drafting are done as part of master's thesis (PS) [17].

### 3.1. Inclusion Criteria

The inclusion criteria were developed using a PICO (problem/population, intervention, comparison, and outcome) framework. The exclusion criteria mirrored the inclusion criteria.

Population: patients with early PD which for the purposes of this study was defined by a short (≤5 years) duration of the disease and a Hoehn and Yahr stage of ≤3. If a study included early and advanced PD patients, then the study was included if there were subanalysis of the patients in the early PD group.

Intervention: All studies that compared either rasagiline or pramipexole to another drug treatment such as placebo or levodopa in patients with early PD were included. Additional PD medication was allowed providing that the doses of the drugs were stable at least for four weeks before the initiation of the study.

Outcome: The unified Parkinson's disease rating scale (UPDRS) total scores at the end of the study or end of study score change from the baseline were extracted from the studies. End of study scores from individual subscales of the UPDRS scale, the UPDRS Part II scores (Motor Aspects of Experiences of Daily Living (M-EDL) or Activities of Daily Living (ADL)), and the UPDRS Part III scores (Motor Examination) were also extracted from the study. Adverse events that appeared in the study population were analyzed by frequency and severity. Adverse events were classified further depending on whether they were related to drug usage, whether they could be classified as severe or serious adverse events, and whether they led to withdrawal of a subject from the study. Severe adverse events were defined as incapacitating or causing inability to work or undertake usual activities, and serious adverse events were classified as life-threatening events which could lead to hospitalization or death or cause prolonged or permanent injury to the patient.

Additional inclusion criteria included the following: (a) only randomized controlled trials were included; (b) study publications had to be in English; and (c) only peer-reviewed studies were included.

### 3.2. Search Strategy

Search strategies were created for PubMed, Cochrane Library, Scopus, Web of Science, PsycINFO, CINAHL, and Medic. All the databases were initially searched in November 2020. The literature search was repeated in September 2021 before the risk of bias analysis and data extraction phase to identify studies that were published during the screening phase. The literature search and the search strategy were created and conducted in collaboration with an information specialist (HL). The search strategy consisted of variations of “early Parkinson's disease,” “pramipexole,” “rasagiline,” “UPDRS”, and “adverse events.” There was language restriction to English and no restriction to time span. The full search strategies for every database are provided in Supplemental Table 1.

### 3.3. Study Selection

**Figure 1:**
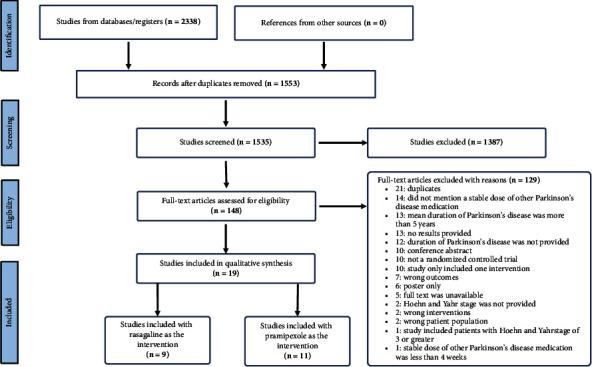
PRISMA flowchart for the literature search process. One included study had both rasagiline and pramipexole as interventions.

Screening, quality assessment, and data extraction were done in Covidence (www.covidence.org). Two reviewers (PS and SB) independently used inclusion criteria to screen titles and abstracts of all the studies that were acquired from the literature search. In case of disagreement, a third reviewer (DCD) resolved the conflict. After title and abstract screening, all included studies went through full-text screening by two independent reviewers (PS and SB). A third reviewer (DCD) resolved all conflicts. See Figure 1 for the PRISMA flowchart. A list of excluded studies in the full-text screening stage, with the reason for exclusion, is provided in Supplemental Table 2.

### 3.4. Data Extraction

One individual (PS) extracted all relevant data from the included studies [17]. General information (first author's name and year), study design, study population characteristics (population size, age, gender, duration of Parkinson's disease, and Hoehn and Yahr stage), study interventions (drug and dosage), duration of the study, endpoint UPDRS total score and UPDRS Part II and III subscores, adverse events, any discontinuation, and whether other PD medications were used during the study were extracted from the studies. A second individual (DCD) double-checked the extracted data. Conflicts were resolved by discussion or by a third reviewer when needed.

### 3.5. Quality Assessment

The methodological quality of each included study was assessed using the Cochrane Collaboration's tool for assessing the risk of bias in randomized trials [18]. This risk of bias tool includes six domains: selection bias, performance bias, detection bias, attrition bias, reporting bias, and other bias. Two authors (PS and SB) independently reviewed all included studies and assigned a value of “high,” “low”, or “unclear” risk of bias to the categories listed above. One reviewer (PS) resolved the conflicts, and another reviewer (DCD) was consulted in case of more complicated conflicts. All decisions were logged using Covidence (https://www.covidence.org/).

Assessment of the quality, quantity, and consistency of evidence across studies was also assessed using the Grading of Recommendations, Assessment, Development, and Evaluation (GRADE) approach [19]. Randomized controlled trials were initially ascribed an initial confidence rating consistent with high-quality evidence (initial score = 4). Several factors were then considered to determine whether this initial rating should be either downgraded or upgraded. Factors that could downgrade the rating included quality, indirectness, inconsistency, and imprecision. Factors that could upgrade the rating included the large magnitude of the effect, dose response, and accounting for plausible confounders. To obtain the final GRADE score for a given outcome, points were deducted from the initial score based on criteria related to the following four categories: quality, directness, consistency, and precision. After a final confidence rating was determined, the rating is translated into a level of evidence using the following scheme: final score ≤1: very low; 2: low; 3: moderate; and ≥4: high. Evidence profiles and summary-of-findings tables were created using a customized form.

### 3.6. Method of Analysis

UPDRS score changes were standardized to a single common measurement which was the mean UPDRS score change from the beginning of the study till the end of the study in our analysis. Combining the scores from each of the studies and comparing the score to the comparator intervention provides a standardized mean difference (SMD). MedCalc version 20.011 was used for statistical analysis. Forest plot analyses were used to evaluate interstudy heterogeneity for main study outcomes. A random-effect, Mantel–Haenszel model (95% CI) was used to determine effect sizes between studies. Statistical heterogeneity was assessed using *I*^2^ statistics: statistically significant *I*^2^ values of >75% represented considerable heterogeneity, *I*^2^ values <40% were deemed unimportant, while intermediate values represented moderate heterogeneity [20]. Heterogeneity was defined in our analysis as differences between study characteristics including population, study length, and other PD medications that were permitted. Random-effect models were preferred over fixed-effect models due to the evidence of high heterogeneity in some analyses. All meta-analyses considered published studies that evaluated main outcomes (change in total UPDRS score, UPDRS Part II score, and UPDRS Part III score).

## 4. Results

### 4.1. Description of the Included Rasagiline Studies

The key characteristics of the nine rasagiline studies are reported in Table 1. One study [21] compared pramipexole and rasagiline as interventions and is included in this portion of the analysis. One post hoc analysis of a randomized controlled trial [22] met the inclusion criteria and was included in the systematic review. There were a total of 2121 patients in the studies, and 1059 patients were on rasagiline: 738 patients had a dose of 1 mg/day, 307 had a dose of 2 mg/day, and 14 had a dose of 4 mg/day. The mean age of study populations ranged from 59.3 to 70.2 years. The mean duration of PD ranged from 2.5 months to 4.8 years. The proportion of the study population who were males in the intervention groups ranged from 42.9 to 76.9%. There were four studies where other PD medications were either discontinued before the study or patients were PD-drug naïve [21, 23–25]. The rest of the studies allowed other PD medications with stable dosage for more than 4 weeks before the start of the study [26–29]. Rescue LD was accepted in one rasagiline study if PD symptoms worsened, and the current therapy did not relieve the symptoms [28]. Hoehn and Yahr stage was ≤3 in every study. The overall discontinuation rates were 7.3% and 10.7% in the rasagiline and placebo groups, respectively. One study reported seven discontinued patients without indicating which intervention the patients were using [27].

### 4.2. Description of the Included Pramipexole Studies

Key characteristics of the 11 pramipexole studies are reported in Table 2. There were a total of 2848 patients in the studies and 1737 patients were on pramipexole. The mean age of study populations ranged from 56.2 to 67.0 years. The mean duration of PD in individual studies ranged from 2.5 months to 4.5 years. The proportion of study population who were males in intervention groups ranged from 47.3 to 69.8%. There were three studies where other PD medications were either discontinued before the study or patients were PD-drug naïve [21, 30, 31]. The rest of the studies permitted other PD medications with stable dosage for more than 4 weeks before the start of the study [32–39]. Rescue LD was accepted in two studies if PD symptoms worsened, and the current therapy did not relieve the symptoms [33, 37]. Hoehn and Yahr stage was ≤3 in every study. The overall discontinuation rates were 18.9% and 17.4% in the pramipexole and placebo groups, respectively.

### 4.3. Quality Assessment and Risk of Bias

**Figure 2:**
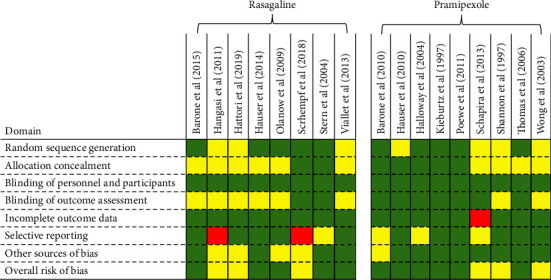
Risk of bias table of the included studies. Green, yellow, and red denote low, unclear, and high risk of bias, respectively.

The risk of bias evaluation is presented in Figure 2. Overall risk of bias for individual criteria is mostly low, but the risk of bias remained unclear for some criteria in some studies. Allocation concealment and blinding of outcome assessment were incompletely reported or unreported in some studies. Blinding of outcome personnel and participants and incomplete outcome data were generally well reported, and no risk of bias associated with these criteria was detected. Risk of bias was mostly low in random sequence generation and in other sources of bias, but in some studies, the risk of bias for these criteria was unclear.

### 4.4. Rasagiline Treatment Efficacy

Seven studies out of nine provided data for UPDRS Part II, Part III, or total scores (Table 3). Change in UPDRS Part II scores ranged in rasagiline groups from 0.78 to −2.17 and in placebo groups from 2.32 to −1.64. Change in UPDRS Part III scores ranged in rasagiline groups from 0.5 to −4.47 and in placebo groups from 2.38 to −2.20. Change in UPDRS total score ranged in rasagiline groups from 1.26 to −3.6 and in placebo groups from 4.27 to −1.2.

**Figure 3:**
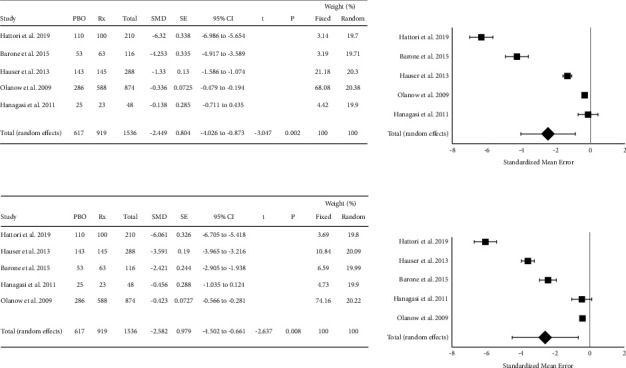
Forest plots of the standardized mean difference in Unified Parkinson's Disease Rating Scale II (UPDRS II (a)) and III (UPDRS III (b)) following the administration of rasagiline at 1 mg/day. Rasagiline improved both UPDRS II and UPDRS scores. Significant heterogeneity was seen in the included studies.

Five studies (*n* = 1536) were included in a meta-analysis comparing the efficacy of rasagiline (1 mg/day) to placebo as determined using UPDRS II and III scores. A random-effect model was used because there was a significant amount of heterogeneity in the study results. Rasagiline significantly improved UPDRS Part II (SMD = −2.449, 95% CI = −4.026 to −0.873, *P*=0.002) and UPDRS Part III compared to placebo (SMD = −2.581, 95% CI −4.502 to −0.661, *P*=0.008). However, there was considerable heterogeneity among the included studies in both analyses (UPDRS Part II: *Q* = 496.6, *P*=0.0001, *I*^2^ = 99.2%; UPDRS Part III: *Q* = 523.5, *P*  <  0.0001, *I*^2^ = 99.2%). Publication bias was not noted for either analysis (Egger's test >0.05). The results from the meta-analysis for UPDRS Parts II and III are presented in Figure 3.

### 4.5. Pramipexole Treatment Efficacy

Ten studies provided data for UPDRS Part II, Part III, or total scores (Table 4). Change in UPDRS Part II scores in the pramipexole treatment group ranged from 0.4 to −3.2 and in comparator groups from 1.5 to −2.2. Change in UPDRS Part III scores in the pramipexole treatment group ranged from 3.4 to −11.5 and in comparator groups from 7.3 to −2.2. Change in UPDRS total score in the pramipexole treatment group ranged from 4.5 to −7.0 and in the comparator groups from 9.2 to −0.9.

**Figure 4:**
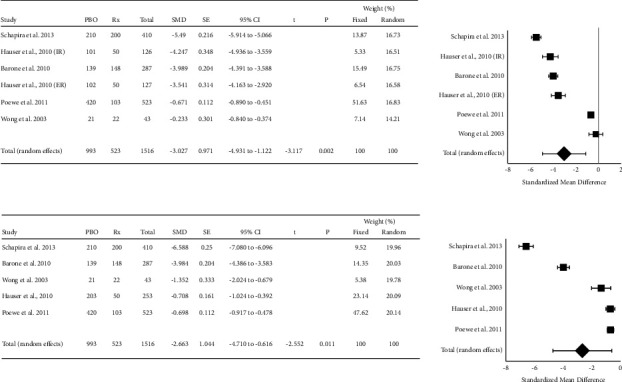
Forest plots of the standardized mean difference in Unified Parkinson's Disease Rating Scale II (UPDRS II (a)) and III (UPDRS III (b)) following the administration of pramipexole. Pramipexole improved both UPDRS II and UPDRS III scores. Significant heterogeneity was seen in the included studies.

Five studies (*n* = 1516) were included in a meta-analysis comparing the efficacy of pramipexole to placebo as determined using UPDRS II and III scores. Data from Hauser and coworkers (2010) [33] were pooled for the analysis of the UPDRS III data. However, UPDRS II data from this study were analyzed by formulation (immediate release (IR) or extended release (ER)) since a significant difference was seen between the groups. In this case, the control group was divided between the two formulations to prevent double counting. Data from Wong et al. [39] reported only subjects receiving placebo or pramipexole. Pramipexole significantly improved UPDRS Part II (SMD = −3.027, 95% CI = −4.931 to −1.122, *P*=0.002) and UPDRS Part III compared to placebo (SMD = −2.663, 95% CI −4.701 to −0.616, *P*=0.011). There was considerable heterogeneity among the included studies in both analyses (UPDRS Part II: *Q* = 592.9, *P*  <  0.0001, *I*^2^ = 99.2%; UPDRS Part III: *Q* = 625.8, *P*  <  0.0001, *I*^2^ = 99.4%). Publication bias was not noted for either analysis (Egger's test >0.05). The results from the meta-analysis for UPDRS Parts II and III are presented in Figure 4.

### 4.6. Relative Risk of Developing an Adverse Event following Rasagiline Treatment

**Figure 5:**
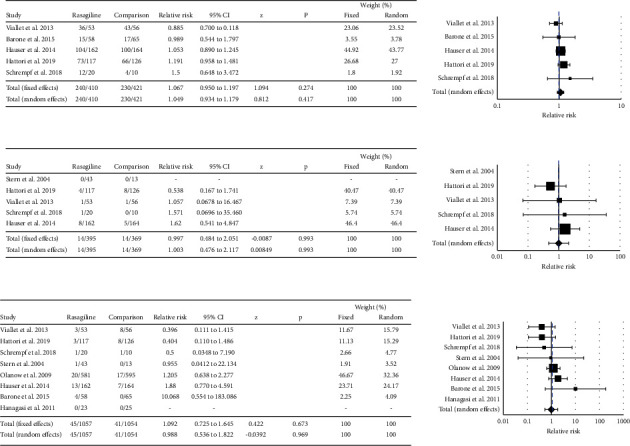
Forest plots of the relative risk of developing any adverse event (a), a serious adverse event (b), or an adverse event leading to withdrawal following the administration of rasagiline (c). Treatment with rasagiline did not increase the incidence of adverse events when compared with the use of either a placebo or pramipexole. The included studies were homogeneous.

All nine studies reported adverse events in patients using rasagiline (listing available in Supplemental Table 3). The frequency of any adverse events, serious adverse events, and adverse events leading to withdrawal were evaluated using meta-analyses (Figure 5). The most common adverse events associated with rasagiline use that were reported across multiple studies included headache (3.4% to 26%), dizziness (5.7% to 23%), nausea (4.2% to 9.4%), back pain (2.6% to 5.1%), and somnolence (0.7% to 6.8%). The study comparing rasagiline and pramipexole reported significantly higher incidences of nausea and vomiting (*P*=0.011) and sleep disorders and daytime sleepiness (*P*=0.027) in patients receiving pramipexole, while rash was more commonly seen in patients receiving rasagiline [21].

The relative risk for developing any adverse event in rasagiline-treated patients did not differ from that seen in placebo- or pramipexole-treated patients (RR = 1.049; 95% CI: 0.934–1.179). Heterogeneity in studies reporting total adverse events following rasagiline administration was low (*Q* = 4.1517, *P*=0.3859, *I*^2^ = 3.65%). The relative risk for developing serious adverse events in rasagiline-treated patients did not differ from that in placebo- or pramipexole-treated patients (RR = 1.003, 95% CI 0.476 to 2.117, *P*=0.993). Heterogeneity in studies reporting serious adverse events was absent (*Q* = 1.8952, *P*=0.5944, *I*^2^ = 0.00%).

Eight studies out of nine (*n* = 2111) were included in a meta-analysis evaluating adverse events that led to withdrawal. The relative risk for developing an adverse event leading to withdrawal in rasagiline-treated patients did not differ from that in placebo- or pramipexole-treated patients (RR = 0.988, 95% CI = 0.536 to 1.822, *P*=0.969). Moderate heterogeneity was detected in studies reporting adverse events resulting in withdrawal (*Q* = 8.7813, *P*=0.1863, *I*^2^ = 31.67%). Publication bias was not noted for any analysis (Egger's test >0.05).

### 4.7. Relative Risk of Developing an Adverse Event following Pramipexole Treatment

**Figure 6:**
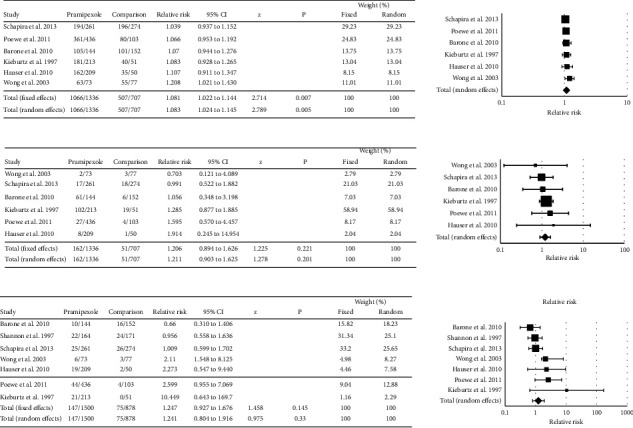
Forest plots of the relative risk of developing any adverse event (a), a serious adverse event (b), or an adverse event leading to withdrawal following the administration of pramipexole (c). Treatment with pramipexole did not increase the incidence of adverse events when compared with the use of either a placebo. The included studies were homogeneous (a, b) or had moderate heterogeneity (c).

All 11 studies reported adverse events in patients receiving pramipexole (listing available in Supplemental Table 4). Incidence rates for the most common adverse events associated with pramipexole monotherapy reported across multiple studies included constipation (5.6 to 20.6%), dizziness (8.3 to 27.4%), fatigue (3.7 to 14.6%), hallucinations (1.9 to 14.6%), headache (4.9% to 20.5%), insomnia (3.5% to 25.8%), nausea (13.9% to 39.0%), and somnolence (8.3% to 36.4%). The frequency of any adverse events, serious adverse events, and adverse events leading to withdrawal was evaluated using meta-analyses (Figure 6). Data for individual studies were pooled across dose groups [36] or dose formulations [33, 37]. Two studies used either levodopa [34, 35] or ropinirole [31] as the comparator, and data from these studies were not included in the meta-analyses.

Six out of 11 pramipexole studies were included in a meta-analysis evaluating the risk of adverse events compared to placebo. The relative risk for developing any adverse events in pramipexole-treated patients was higher than that seen in placebo-treated patients (RR = 1.083; 95% CI: 1.024–1.145, *P*=0.005). Studies reporting total adverse events following pramipexole administration were homogeneous (*Q* = 2.399, *P*=0.7916, *I*^2^ = 0.00%). The relative risk for developing serious adverse events in pramipexole-treated patients did not differ from that in placebo-treated patients (RR = 1.211, 95% CI 0.903 to 1.625, *P*=0.201). Studies reporting serious adverse events were homogeneous (*Q* = 1.3593, *P*=0.9287, *I*^2^ = 0.00%). Seven out of 11 studies (*n* = 2378) were included in a meta-analysis evaluating adverse events that led to withdrawal. The relative risk for developing an adverse event leading to withdrawal in pramipexole-treated patients did not differ from that in placebo- or pramipexole-treated patients (RR = 1.247, 95% CI = 0.927 to 1.676, *P*=0.145). Moderate heterogeneity was detected in studies reporting adverse events resulting in withdrawal (*Q* = 9.8564, *P*=0.1308, *I*^2^ = 39.13%). Publication bias was also present for this collection of studies (Egger's test; intercept = 2.08, 95% CI = 0.2385 to 3.9279, *P*=0.0337).

### 4.8. Rating the Overall Quality of Evidence

Overall evidence from experimental studies was evaluated using GRADE. All studies received an initial score of four. The level of evidence was subsequently downgraded once due to a lack of consistency due to considerable heterogeneity. The calculated confidence intervals in the meta-analyses were relatively broad, and this was likely due to inconsistency rather than imprecision; thus, no additional downgrade for imprecision was applied. Neither risk of bias nor publication bias resulted in a downgrade. No upgrades for large magnitude of effect, dose-response gradient, and residual confounding were deemed likely to decrease the magnitude of the effect. The summary of findings for the main outcomes is provided in Table 5.

## 5. Discussion

Our study showed that both rasagiline (at 1 mg/day) and pramipexole were effective in significantly improving UPDRS Part II and III scores when compared to results seen in patients taking a placebo. Our results showing the benefit of rasagiline on UPDRS Parts II and III are similar to those reported in a systematic review and meta-analysis by Chang et al. [12]. Chang et al. [12] included 10 studies showing rasagiline treatment significantly improved UPDRS Part I, Part II, and Part III scores in PD patients when compared to placebo, but this meta-analysis was not restricted to patients with early PD. In parallel with our study, they also found a significant amount of heterogeneity among UPDRS Part II with rasagiline dose of 1 mg/day (*Q* = 13.9, *P*=0.031, *I*^2^ = 56.8%) and 2 mg/day (*Q* = 5.5, *P*=0.019, *I*^2^ = 81.9%) [12]. Hauser et al. [40] conducted a meta-analysis on two studies [24, 41] that assessed the efficacy of rasagiline in early PD patients. Rasagiline improved total UPDRS scores ≥3 units and there was significant improvement in UPDRS Part II and Part III scores following rasagiline administration [40]. A meta-analysis by Mínguez-Mínguez et al. [42] showed decreases in total UPDRS score was −3.06 units (95% CI −2.31 to −3.81, *P*  <  0.00001) and −3.17 units (95% CI −3.91 to −2.42, *P*  <  0.00001) in patients receiving rasagiline at either 1 or 2 mg/day. Our results regarding the safety of rasagiline are parallel with other meta-analyses showing rasagiline to have similar safety profiles to placebo [12, 13, 42, 43].

When compared to rasagiline, fewer systematic reviews and meta-analyses are available for pramipexole and PD patients. A recent systematic review by Chen et al. [44] identified six trials that compared the efficacy of pramipexole versus placebo in patients with early PD as defined by a Hoehn and Yahr score of <3. This study showed no benefit of pramipexole at 22 to 30 weeks after initiation of therapy on the change in either UPDRS Part II (mean difference = 0.02, 95% confidence interval: −1.15 to 1.12) or Part III (mean difference = 0.32, 95% confidence interval: −8.22 to 7.95) scores. Evaluation of safety data showed that pramipexole demonstrated significantly higher event rates for nausea than placebo [44]. A systematic review by Ji et al. [45] including 23 randomized clinical trials showed pramipexole was effective in lowering the Hamilton Depression Rating Scale (HAM-D) score in PD patients with anxiety or depression. The incidence of adverse events was lower in PD patients treated with pramipexole when compared with controls. Wang et al. [15] evaluated the efficacy and safety of pramipexole and levodopa combination therapy versus levodopa monotherapy in patients with PD. In the meta-analysis, pramipexole and levodopa combination therapy improved the motor UPDRS score (SMD −1.31, 95% CI −1.57 to −1.04, *P*  <  0.00001), the UPDRS score for activities of daily living (SMD −1.26, 95% CI −1.49 to −1.03, *P*  <  0.00001), the UPDRS score for mental activities (SMD −1.02, 95% CI -1.27 to −0.77, *P*  <  0.00001), and the UPDRS score for complications (SMD −1.54, 95% CI −1.93 to −1.15, *P*  <  0.00001) [15]. In parallel with our study, significant heterogeneity was observed in the UPDRS score analysis. In contrast to levodopa monotherapy, pramipexole and levodopa combination therapy reduced the number of any adverse events in PD patients (OR 0.36, 95% CI 0.27 to 0.50, *P*  <  0.00001).

There was a significant amount of heterogeneity in our meta-analyses which we attribute to different study characteristics including study lengths, study population characteristics, and other treatments that were permitted. This resulted in a moderate level of confidence in our findings for both therapies. Study designs also varied; for example, one included study [21] compared the efficacy of rasagiline versus pramipexole rather than placebo. Likewise, two pramipexole studies used either levodopa [34, 35] or ropinirole [31] rather than a placebo as the comparator. Studies that used a comparator other than a placebo were excluded from our meta-analyses, limiting our ability to draw conclusions regarding their efficacy.

In our systematic review, the definition for early PD is based on Hoehn and Yahr stage of I–III and the duration of PD being ≤5 years. Disease duration and staging using the Hoehn and Yahr scale remain difficult since PD patients often have variable disease progression, inconsistent severity of symptoms, the possibility of genetic mutations affecting the disease, environmental and lifestyle factors that affect the disease, and premotor and prodromal stages that can affect decades before the appearance of motor symptoms [46]. These factors may contribute to the high degree of heterogeneity observed. Some retrieved studies that were excluded failed to mention whether stable doses of other drugs were achieved. Restricting our review to studies published in English is another factor to take into account considering the number of clinical trials performed in China (see a meta-analysis by Ji et al. [45]).

In conclusion, rasagiline and pramipexole significantly improved UPDRS Part II and III scores when compared to placebo. Neither rasagiline nor pramipexole increased the relative risk for any adverse events, serious adverse events, or adverse events leading to withdrawal when compared with placebo.

## Figures and Tables

**Table 1:** The main characteristics of the rasagiline studies included in the systematic review.

Study, year	Intervention	Dose (mg/day)	Duration (wk.)	Population (*n*)	Number of withdrawals	Mean age (yr.)	Males (%)	Duration of PD (yr.)	H&Y stage	Country	Other PD medications in entire population (%)
Barone et al. 2015	RA vs. PBO	1	12	65	8	66.1 ± 8.4	58.5	4.8 ± 3.8	1.9 ± 0.5	Italy	LD (65.8), LD + CD + EC (15.4), Me-LD (8.9), RR (13.8), PPX (40.1), RG (6.5), EC (1.6)
				58	9	66.0 ± 8.7	46.6	3.7 ± 3.2	1.8 ± 0.5		

Hanagasi et al. 2011	RA vs. PBO	1	12	25	7 (PBO + RA)^a^	67.6 ± 10.1	64.0	4.0 ± 2.3	1.6 ± 0.6	Turkey	LD and derivatives (91.7), DAA (54.2)
				23 7 (dropouts)		65.2 ± 9.5	73.9	4.1 ± 2.5	2.0 ± 0.7		
Hattori et al. 2019	RA vs. PBO	1	26	126	26	65.4 ± 8.8	42.9	1.6 ± 1.2	2.2 ± 0.6	Japan	Other PD medications were discontinued >30 day before the study
				118	8	67.4 ± 9.0	44.9	2.0 ± 2.0	2.2 ± 0.6		

Hauser et al. 2014^b^	RA vs. PBO	1	18	165	20	62.8 ± 10.1	68.5	2.1 ± 1.9	≤3	United States	DAA (100) (PPX (58.6), RR (41.4)), AM (6.7), AC (3.7), rescue LD (3.4)
				163	20	62.3 ± 9.3	67.9	2.2 ± 2.2	≤3		

Olanow et al. 2009	RA vs. PBO	1, 2	72	595^d^	50^e^	62.2 ± 9.7	61.8	4.4 ± 4.6 mo.	1.5 ± 0.5	14 countries	The study included only subjects who were not receiving any PD treatment
Rascol et al. 2011^c^				288 293	15 20	62.4 ± 9.7 62.3 ± 9.6	60.8 59.7	4.6 ± 4.7 mo. 4.6 ± 4.6 mo.	1.5 ± 0.5 1.5 ± 0.5		

Schrempf et al. 2018	RA vs. PBO	1	8	10	3	70.2 ± 7.3	60.0	3.3 ± 3.5	2.3 ± 0.8	Germany	LD (53.3), DAA (60.0), COMT-I (3.3)
				20	2	69.9 ± 6.9	50.0	4.0 ± 3.5	1.9 ± 0.8		

Stern et al. 2004	RA vs. PBO	1, 2, 4	10	13	0	64.8 ± 9.4	76.9	0.8 ± 1.0	1.5 ± 0.4	United States	Other PD drugs not permitted
				15	1	59.3 ± 8.6	66.7	1.3 ± 2.6^f^	1.5 ± 0.4		
				14	0	60.3 ± 7.2	71.4	0.4 ± 0.8	1.6 ± 0.4		
				14	0	62.0 ± 9.7	57.1	0.3 ± 0.5	1.6 ± 0.4		

Viallet et al. 2013	PPX vs. RA	1.5	15	56	14	62.1 ± 6.2	55.4	4.3 ± 7.3 mo.	≤3^g^	France	All PD treatments were discontinued
		1		53	3	63.2 ± 7.3	69.8	2.5 ± 3.8 mo.	≤3^g^		

Values are reported as mean ± standard deviation. CD = carbidopa, DAA = dopamine agonists, EC = entacapone, mo = months, PBO = placebo, PD = Parkinson's disease, PPX = pramipexole, RA = rasagiline, RCT = randomized controlled trial, RG = rotigotine, RR = ropinirole, AC = anticholinergics, AM = amantadine, COMT-I = Catechol-O-methyl transferase inhibitors, LD = levodopa, MAOB-I = monoamine oxidase B inhibitors, wk = weeks, yr = years, and H&Y stage = Hoehn and Yahr stage. ^a^Withdrawals were due to noncompliance. ^b^Rasagiline as add-on therapy to dopamine agonists in patients with early PD. ^c^Post hoc analysis of the ADAGIO study [24]. ^d^Delayed-start rasagiline 1 mg and 2 mg groups are combined as one because the treatment was in both placebo and we extracted data from week 36. ^e^Withdrawals reported before starting active treatment at 36 weeks. ^f^One patient was reported as having a disease duration of 10 years. ^g^94% of study subjects had a Hoehn and Yahr stage of ≤2.

**Table 2:** The main characteristics of the pramipexole studies included in the systematic review.

Study, year	Intervention	Dose (mg/day)^a^	Duration	Population (*n*)	Number of withdrawals	Mean age ± SD (yr.)	Males (%)	Duration of PD (yr.)	H&Y stage	Country	Other PD medications in entire population (%)
Barone et al. 2010	PPX vs. PBO	0.375–3 (2.18 ± 0.83)	12 wk	152	19	66.6 ± 9.9	51.3	4.5 ± 3.9	2.1 ± 0.6	12 European countries, South Africa	LD and derivatives^b^ (74.7), AM (23.3), MAOB-I (13.2), AC (7.8), other (5.4)^c^
				144	20	67.4 ± 9.0	43.1	4.0 ± 4.5	2.2 ± 0.6		

Hauser et al. 2010	PPX ER or PPX IR vs. PBO	0.375–4.5	18 wk	50	4	63.2 ± 8.7	46.0	0.8 ± 1.1	1–3	14 countries, Europe, US, South America, Asia	MAOB-I, AM, AC, *β*-blockers. Rescue LD 14.0 (PBO), 2.9 (ER), 1.0 (IR)
		3.05 ± 1.37 (ER)		106	21	61.6 ± 9.4	58.5	1.1 ± 1.3	1–3		
		3.03 ± 1.39 (IR)		103	15	62.0 ± 8.3	57.3	0.9 ± 1.2	1–3		

Parkinson Study Group 2000	PPX vs. LD	300–600 (LD)^e^ (LD: 427 ± 112)	48 mo.	150	49	60.8 ± 9.8	68.0	1.8 ± 1.7	1.8 ± 0.4	US, Canada	Open-label LD 59.3 (LD) and 72.2 (PPX), EL (35.5), AM (25.2), AC (9.6), COMT-I (2.3)^f^
Parkinson Study Group 2004^d^		0.75–4.5 (PPX) (PPX: 2.8 ± 1.1)		151	67	61.1 ± 9.6	60.2	1.5 ± 1.4	1.9 ± 0.4		

Kieburtz et al. 1997	PPX vs. PBO	1.5–6.0	10 wk	51	0	60.4 ± 12.0	62.7	1.7 ± 1.5	1.8 ± 0.5	US	AC, AM, and SE were permitted with stable dose for 30 days prior to the study. SE use 55.6–68.0%
				54	10	60.3 ± 10.5	64.8	1.8 ± 1.5	1.8 ± 0.6		
				50	2	62.2 ± 11.1	62.0	2.0 ± 1.6	1.9 ± 0.5		
				54	4	62.8 ± 10.5	63.0	1.9 ± 1.5	1.8 ± 0.5		
				55	9	62.8 ± 11.4	69.1	2.2 ± 1.8	1.9 ± 0.6		

Poewe et al. 2011	PPX ER or PPX IR vs. PBO	0.375–4.5	33 wk	103	12	62.0 ± 9.6	49.5	0.9 ± 1.0	2.1 ± 0.6	14 countries Europe, US, South America, Asia	In each group AM (29.6%–31.5%) MAOB-I (25.2%–29.1%), AC (20.2%–21.5%), rescue LD (7.0 ER, 4.3 IR, 21.4 PBO)
		2.9 ± 1.4 (ER)		223	49	61.3 ± 9.8	57.0	1.0 ± 1.2	1.6 ± 0.6		
		2.9 ± 1.4 (IR)		213	37	61.7 ± 9.6	56.8	1.1 ± 1.4	2.1 ± 0.6		

Schapira et al. 2013	PPX vs. PBO	1.5	15 mo.	274	60^g^	62.9 ± 9.9	61	4.5 ± 5.9 mo.	1.5 ± 0.5	10 countries Europe, US, Asia	Other PD medications were not permitted
				261	40	62.1 ± 10.1	68	4.4 ± 6.3 mo.	1.5 ± 0.4		

Shannon et al. 1997	PPX vs. PBO	0.375–4.5 (3.8)	31 wk	171	34	62.7 (PBO + PPX)	60.6 (PBO + PPX)	1.8 (PBO + PPX)	1–3	NR	SE with stable dosage (about two-thirds in each group)
				164	28						

Thomas et al. 2006	PPX vs. RR	2.1–4.2	24 mo.	30	3	57.1 ± 2.0^h^	56.0	1.2 ± 0.5 (PPX + RR)	1.6 ± 0.6	Italy	Patients had never received any PD treatment
		15–24		30	5	55.3 ± 2.0	55.6		1.4 ± 0.6		

Viallet et al. 2013	PPX vs. RA	1.5 (PPX)	15	56	14	62.1 ± 6.2	55.4	4.3 ± 7.3 mo.	≤3^i^	France	All PD treatments were discontinued
		1 (RA)		53	3	63.2 ± 7.3	69.8	2.5 ± 3.8 mo.	≤3^i^		

Wong et al. 2003	PPX vs. PBO	0.375–4.5	15	77	8	60.9 ± 1.1	72.7	4.3 ± 0.4	2.2 ± 0.1	Hong Kong, Taiwan	Stable LD, SE, AC, AM
				73	9	58.8 ± 1.3	65.8	4.5 ± 0.4	2.2 ± 0.1		

AC = anticholinergics, AM = amantadine, COMT-I = Catechol-O-methyl transferase inhibitors, EL = eldepryl, ER = extended release, IR = immediate release, H&Y = Hoehn and Yahr stage, LD = levodopa, MAOB-I = monoamine oxidase B inhibitors, mo = months, NR = not reported, PBO = placebo, PD = Parkinson's disease, PPX = pramipexole, RA = rasagiline, RCT = randomized controlled trial, RR = ropinirole, SE = selegiline, US = the United States of America, wk = weeks, and yr = years. ^a^In most studies, doses were titrated at the beginning of the study. Mean values in brackets. ^b^Levodopa with or without carbidopa, levodopa plus carbidopa plus entacapone, or levodopa plus benserazide. ^c^Entacapone or budipine. ^d^The first 2 yr. are reported in Parkinson Study Group [34] and whole 4 yr. study is reported in Parkinson Study Group [35]. ^e^Carbidopa and levodopa preparation. First value is the amount of carbidopa and the second one is the amount of levodopa. ^f^Values reported of the whole study population (*n* = 301). ^g^All subjects that discontinued the study before active treatment at 9 months. This study had a delayed-start design that used placebo until 9 months when patients were switched to placebo. We are reporting the data before the switch to an active treatment. PBO group means in this table the delayed-start pramipexole group. ^h^The results are reported of patients that completed the study. ^i^94% of study subjects had a H&Y score of ≤2.

**Table 3:** Change in UPDRS scores seen following rasagiline administration.

Change in UPDRS						
Study, year	Time of assessment (from the start of the study)	Treatment	Part II	Part III	Total	Comments
Barone et al. 2015	12 wk.	PBO	0.06 ± 0.32	0.42 ± 0.51	NR	—
		RA	**−1.37** **±** **0.35** (**P**=0.003)	−0.88 ± 0.56 (*P*=0.09)	NR	

Hanagasi et al. 2011	12 wk.	PBO	−1.64 ± 3.59	−2.20 ± 4.05	NR	—
		RA	−2.17 ± 3.95 (*P*=0.539)	−4.35 ± 5.21 (*P*=0.116)	NR	

Hattori et al. 2019	26 wk.	PBO	2.32 ± 0.34	−0.48 ± 0.64	NR	—
		RA	**0.13** **±** **0.35** (**P** < 0.0001)	**−4.47** **±** **0.67** (**P** < 0.0001)	NR	

Hauser et al. 2014	18 wk.	PBO	0.3 ± 0.3	−1.6 ± 0.5	−1.2 ± 0.7	—
		RA	−0.1 ± 0.3 (*P*=0.3)	**−3.4** **±** **0.5** (**P**=0.007)	**−3.6** **±** **0.7** (**P**=0.012)	

Olanow et al. 2009	36 wk.	PBO	1.64 (1.43 to 1.85)	2.38 (1.96 to 2.79)	**4.27** **±** **0.26** (**P** < 0.001)	Estimates of change from baseline in UPDRS subscores at week 36. *N* = 588 (PBO), *N* = 286 (RA 1), *N* = 290 (RA 2)
Rascol et al. 2011^a^		RA 1 mg RA 2 mg	0.78 (0.49 to 1.06) **−0.86** **±** **0.18** (**P** < 0.0001) 0.76 (0.47 to 1.04) **−0.88** **±** **0.18** (**P** < 0.0001)	0.50 (−0.07 to 1.07) **−1.88** **±** **0.35** (**P** < 0.0001) 0.20 (−0.37 to 0.76) **−2.18** **±** **0.35** (**P** < 0.0001)	1.26 ± 0.36 1.11 ± 0.36	

Schrempf et al. 2018	8 wk.	PBO	NR	NR	NR	*N* = 17 (RA). PBO data NR
		RA	NR	−2.0 ± 6.2 (*P*=0.205)	−0.7 ± 7.6 (*P*=0.708)	

Stern et al. 2004	10 wk.	PBO	NR	NR	−0.5 ± 0.8	“Repeated measures analysis for the improvement in total UPDRS score during the 10-week period showed a significant change (*P* < 0.05) for the RA 2 mg group but not for the 1 and 4 mg groups, compared with PBO”
		RA 1 mg	NR	NR	−1.8 ± 1.3	
		RA 2 mg	NR	NR	**−3.6** **±** **1.7**	
		RA 4 mg	NR	NR	−3.6 ± 1.2	

Values reported as mean ± standard deviation. NR = not reported, PBO = placebo, RA = rasagiline, wk = week, UPDRS = the unified Parkinson's disease rating scale. ^a^Post hoc analysis of the ADAGIO study [24]. Statistically significant results are highlighted using bold text.

**Table 4:** Change in UPDRS scores seen following pramipexole administration.

Change in UPDRS						
Study, year	Time of assessment (from the start of the study)	Treatment	Part II	Part III	Total	Comments
Barone et al. 2010	5 wk.	PBO	NR	−1.0 ± 1.0	NR	UPDRS score had different number of patients PBO (*n* = 148), PPX (*n* = 139)
		PPX	NR	**−4.1** **±** **1.0** (**P**=0.003)	NR	
	12 wk.	PBO	−1.2 ± 0.3	−2.2 ± 0.5	NR	
		PPX	**−2.4** **±** **0.3** (**P**=0.003)	**−4.4** **±** **0.3** (**P**=0.003)	NR	

Hauser et al. 2010	18 wk.	PBO	0.0 ± 0.5	−2.7 ± 1.0	NR	UPDRS scores had different number of patients PBO (*n* = 50), ER (*n* = 102), IR (*n* = 101). Levodopa data censored in the scores
		PPX ER	**−1.5** **±** **0.4** (**P**=0.0023)	**−5.9** **±** **0.9** (**P**=0.0039)	NR	
		PPX IR	**−1.8** **±** **0.4** (**P**=0.0005)	**−5.9** **±** **0.8** (**P**=0.0038)	NR	

Parkinson Study Group 2000^a^	23.5 mo.	PPX	1.1 ± 4.5	3.4 ± 8.6	4.5 ± 12.7	Values are reported from the whole study population (*n* = 301). Negative values indicated worsening and positive values indicated improvement
		LD	**2.2** **±** **3.2** (**P**=0.001)	**7.3** **±** **8.6** (**P** < 0.001)	**9.2** **±** **10.8** (**P** < 0.001)	
Parkinson Study Group 2004	48 mo.	PPX	−1.7 ± 5.4	−1.3 ± 13.3	−3.2 ± 17.3	
		LD	**−0.5** **±** **4.7** (**P**=0.02)	**3.4** **±** **12.3** (**P**=0.001)	**2.0** **±** **15.4** (**P**=0.003)	

Kieburtz et al. 1997	10 wk.	PBO	NR	NR	−0.9 ± 9.1	The score in the parenthesis: secondary analysis of changes from baseline to 10 weeks in total UPDRS score. Difference between treatment group mean and placebo group mean. *P* < 0.001 for all doses
		PPX 1.5	NR	NR	−6.3 ± 9.0 **(-5.24 (−8.95 to −1.54))**	
		PPX 3.0	NR	NR	−5.9 ± 6.4 **(-5.08 (−8.86 to −1.29))**	
		PPX 4.5	NR	NR	−6.5 ± 8.2 **(-5.86 (−9.59 to −2.13))**	
		PPX 6.0	NR	NR	−7.0 ± 8.1 **(-5.24 (−8.96 to −1.53))**	

Poewe et al. 2011	33 wk.	PBO	−0.2 (−0.9 to 0.4)	−1.1 (−2.5 to 0.3)	NR	*N* = 103 (PBO), *N* = 213 (ER), *N* = 207 (IR)
		PPX ER	**−2.1 (−2.5 to −1.6) ** (**P** < 0.0001)	**−6.1 (−7.1 to −5.1) ** (**P** < 0.0001)	NR	
		PPX IR	**−2.4 (−2.8 to −1.9) ** (**P** < 0.0001)	**−6.4 (−7.4 to −5.4) ** (**P** < 0.0001)	NR	

Schapira et al. 2013	6 to 9 mo.^b^	PBO	1.5 ± 0.2	2.7 ± 0.5	4.3 ± 0.6	*N* = 200 (PBO), *N* = 210 or 211 depending on time points (PPX)
		PPX	0.4 ± 0.2 **−1.1 (−1.7 to −0.5)** (**P** < 0.0001)	−0.6 ± 0.5 **−3.3 (−4.5 to −2.2)** (**P** < 0.0001)	−0.5 ± 0.6 **−4.8 (−6.3 to −3.2)** (**P** < 0.0001)	

Shannon et al. 1997	31 wk.	PBO	0.4	1.3	NR	—
		PPX	**−1.8 ** (**P** < 0.0001)	**−4.7 ** (**P** < 0.0001)	NR	

Thomas et al. 2006	24 mo.	PPX	NR	11.9 ± 2.3	NR	—
		RR	NR	12.6 ± 2.8	NR	

Wong et al. 2003	15 wk. without LD	PBO	−2.2 ± 0.6	−1.3 ± 1.6	NR	Patients without levodopa (*n* = 22 PBO), (*n* = 21, PPX)
		PPX	−2.9 ± 0.6 (*P*=0.2435)	**−11.5** **±** **1.6** (**P**=0.0110)	NR	
	15 wk. with LD	PBO	−0.5 ± 0.5	−0.6 ± 1.1	NR	Patients with levodopa (*n* = 54 PBO), (*n* = 50, PPX)
		PPX	**−3.2** **±** **0.5** (**P**=0.0003)	**−7.2** **±** **1.3** (**P**=0.0001)	NR	

Values are reported as mean ± standard deviation. ER = extended release, IR = immediate release, LD = levodopa, mo = months, NR = not reported, PBO = placebo PPX = pramipexole, UPDRS = the unified Parkinson's disease rating scale, RR = ropinirole, and wk = weeks. ^a^Parkinson Study Group [34] reported the first 2 yr. of the study and Parkinson Study Group [35] reported results at the end of the 4-year study. ^b^Patients were assigned to the pramipexole group at 9 months or as early as 6 months, if the patients expressed inability to tolerate PD symptoms. Statistically significant results are highlighted using bold text.

**Table 5:** Summary of findings.

Outcome	Treatment	Number of studies	Quality of the evidence (GRADE)	Conclusion
Change in UPDRS II	Rasagiline	5 (5)	Moderate	Possible benefit; however, additional research is needed
	Pramipexole	7 (5)	Moderate	
Change in UPDRS III	Rasagiline	5 (5)	Moderate	
	Pramipexole	7 (5)	Moderate	

Values in parentheses represent the number of studies that contributed to the meta-analyses. UPDRS = the unified Parkinson's Disease rating scale. GRADE = grading of recommendations, assessment, development, and evaluations.

## Data Availability

The data that support the findings of this study are available from the corresponding author upon reasonable request.

## Supplementary Materials

Supplementary Materials Supplemental Table 1: search strategies for databases. The table includes the search strategy of the literature. Supplemental Table 2: explanation for exclusion of study in full-text screening section. The table describes exclusion criteria in full-text screening. Supplemental Table 3: adverse events reported in patients receiving rasagiline. The table includes studies reporting adverse events in patients using rasagiline. Supplemental Table 4: adverse events reported in patients receiving pramipexole. The table includes studies reporting adverse events in patients using pramipexole.
