# Supplementary material for: A Systematic Review and Meta-Analysis of the Efficacy and Safety of Rasagiline or Pramipexole in the Treatment of Early Parkinson's Disease
Source: Parkinsons Dis. 2024 Jan 16;2024:8448584. doi: 10.1155/2024/8448584 (PMC10805557; doi:10.1155/2024/8448584)
Supplement: Supplementary Materials — Supplemental Table 1: search strategies for databases. The table includes the search strategy of the literature. Supplemental Table 2: explanation for exclusion of study in full-text screening section. The table describes exclusion criteria in full-text screening. Supplemental Table 3: adverse events reported in patients receiving rasagiline. The table includes studies reporting adverse events in patients using rasagiline. Supplemental Table 4: adverse events reported in patients receiving pramipexole. The table includes studies reporting adverse events in patients using pramipexole. [file 8448584.f1.docx]

Supplemental Table 1. Search strategies for databases

PubMed Advanced search

#1 "Parkinson Disease"[mh] OR "Parkinson Disease, Secondary"[mh]

#2 "parkinson disease"[tw] OR "parkinson's disease"[tw] OR parkinsonism*[tw]

#3 #1 OR #2

#4 Pramipexole[mh] OR rasagiline[tw]

#5 pramipexol*[tw] OR dexpramipexol*[tw] OR mirapex[tw] OR sifrol*[tw] OR "KNS 760704"[tw] OR KNS760704[tw] OR "SND 919"[tw] OR "SND 919CL2x"[tw] OR SND919[tw] OR SND919CL2x[tw] OR azilect[tw] OR "AGN 1135"[tw] OR AGN1135[tw] OR "TVP 101"[tw] OR TVP101[tw] OR "TVP 1022"[tw] OR TVP1022[tw]

#6 #4 OR #5

#7 #3 AND #6

#8 UPDRS [tw] OR "unified parkinson's disease rating scale"[tw] OR "unified parkinson disease rating scale"[tw]

#9 "Drug-Related Side Effects and Adverse Reactions"[mh]

#10 "adverse effect*"[tw] OR "adverse event*"[tw] OR "adverse reaction*"[tw] OR "adverse drug effect*"[tw] OR "adverse drug event*"[tw] OR "adverse drug reaction*"[tw] OR "side effect*"[tw] OR "injurious effect*"[tw] OR "injurious event*"[tw] OR "injurious reaction*"[tw] OR "ill effect*"[tw] OR "ill event*"[tw] OR "ill reaction*"[tw] OR harm[tw] OR harms[tw] OR harmful*[tw] OR safety[tw] OR toxic*[tw]

#11 #8 OR #9 OR #10

#12 #7 AND #11

#13 "Randomized Controlled Trials as Topic"[mh] OR "Random Allocation"[mh] OR "Randomized Controlled Trial"[pt] OR "Clinical Trials as Topic"[mh] OR "Clinical Trial"[pt] OR "Double-Blind Method"[mh] OR Placebos[mh]

#14 rct[tw] OR rcts[tw] OR "randomized controlled"[tw] OR "randomised controlled"[tw] OR "random control"[tw] OR "randomized trial*"[tw] OR "randomised trial*"[tw] OR "random allocation"[tw] OR randomly[tw] OR "clinical trial*"[tw] OR "controlled trial*"[tw] OR "control trial*"[tw] OR "double blind*"[tw] OR "double mask*"[tw] OR placebo*[tw]

#15 #13 OR #14

#16 #12 AND #15

#17 #16 AND english[la]

Cochrane Library

#1 ("parkinson disease" OR "parkinson's disease" OR parkinsonism*):ti,ab,kw

#2 (pramipexol* OR rasagiline OR dexpramipexol* OR mirapex OR sifrol* OR "KNS 760704" OR KNS760704 OR "SND 919" OR "SND 919CL2x" OR SND919 OR SND919CL2x OR azilect OR "AGN 1135" OR AGN1135 OR "TVP 101" OR TVP101 OR "TVP 1022" OR TVP1022):ti,ab,kw

#3 (UPDRS OR "unified parkinson's disease rating scale" OR "unified parkinson disease rating scale" OR "adverse effect*" OR "adverse event*" OR "adverse reaction*" OR "adverse drug effect*" OR "adverse drug event*" OR "adverse drug reaction*" OR "side effect*" OR "injurious effect*" OR "injurious event*" OR "injurious reaction*" OR "ill effect*" OR "ill event*" OR "ill reaction*" OR harm OR harms OR harmful* OR safety OR toxic*):ti,ab,kw

#4 #1 AND #2 AND #3

Scopus Advanced search

#1 TITLE-ABS-KEY("parkinson disease" OR "parkinson's disease" OR parkinsonism*)

#2 TITLE-ABS-KEY(pramipexol* OR rasagiline OR dexpramipexol* OR mirapex OR sifrol* OR "KNS 760704" OR KNS760704 OR "SND 919" OR "SND 919CL2x" OR SND919 OR SND919CL2x OR azilect OR "AGN 1135" OR AGN1135 OR "TVP 101" OR TVP101 OR "TVP 1022" OR TVP1022)

#3 TITLE-ABS-KEY(UPDRS OR "unified parkinson's disease rating scale" OR "unified parkinson disease rating scale" OR "adverse effect*" OR "adverse event*" OR "adverse reaction*" OR "adverse drug effect*" OR "adverse drug event*" OR "adverse drug reaction*" OR "side effect*" OR "injurious effect*" OR "injurious event*" OR "injurious reaction*" OR "ill effect*" OR "ill event*" OR "ill reaction*" OR harm OR harms OR harmful* OR safety OR toxic*)

#4 TITLE-ABS-KEY(rct OR rcts OR "randomized controlled" OR "randomised controlled" OR "random control" OR "randomized trial*" OR "randomised trial*" OR "random allocation" OR randomly OR "clinical trial*" OR "controlled trial*" OR "control trial*" OR "double blind*" OR "double mask*" OR placebo*)

#5 #1 AND #2 AND #3 AND #4

Web of Science

#1 TS=("parkinson disease" OR "parkinson's disease" OR parkinsonism*)

#2 TS=(pramipexol* OR rasagiline OR dexpramipexol* OR mirapex OR sifrol* OR "KNS 760704" OR KNS760704 OR "SND 919" OR "SND 919CL2x" OR SND919 OR SND919CL2x OR azilect OR "AGN 1135" OR AGN1135 OR "TVP 101" OR TVP101 OR "TVP 1022" OR TVP1022)

#3 TS=(UPDRS OR "unified parkinson's disease rating scale" OR "unified parkinson disease rating scale" OR "adverse effect*" OR "adverse event*" OR "adverse reaction*" OR "adverse drug effect*" OR "adverse drug event*" OR "adverse drug reaction*" OR "side effect*" OR "injurious effect*" OR "injurious event*" OR "injurious reaction*" OR "ill effect*" OR "ill event*" OR "ill reaction*" OR harm OR harms OR harmful* OR safety OR toxic*)

#4 TS=(rct OR rcts OR "randomized controlled" OR "randomised controlled" OR "random control" OR "randomized trial*" OR "randomised trial*" OR "random allocation" OR randomly OR "clinical trial*" OR "controlled trial*" OR "control trial*" OR "double blind*" OR "double mask*" OR placebo*)

#5 #1 AND #2 AND #3 AND #4

PsycINFO (EBSCOhost)

S1 "parkinson disease" OR "parkinson's disease" OR parkinsonism*

S2 pramipexol* OR rasagiline OR dexpramipexol* OR mirapex OR sifrol* OR "KNS 760704" OR KNS760704 OR "SND 919" OR "SND 919CL2x" OR SND919 OR SND919CL2x OR azilect OR "AGN 1135" OR AGN1135 OR "TVP 101" OR TVP101 OR "TVP 1022" OR TVP1022

S3 UPDRS OR "unified parkinson's disease rating scale" OR "unified parkinson disease rating scale" OR "adverse effect*" OR "adverse event*" OR "adverse reaction*" OR "adverse drug effect*" OR "adverse drug event*" OR "adverse drug reaction*" OR "side effect*" OR "injurious effect*" OR "injurious event*" OR "injurious reaction*" OR "ill effect*" OR "ill event*" OR "ill reaction*" OR harm OR harms OR harmful* OR safety OR toxic*

S4 rct OR rcts OR "randomized controlled" OR "randomised controlled" OR "random control" OR "randomized trial*" OR "randomised trial*" OR "random allocation" OR randomly OR "clinical trial*" OR "controlled trial*" OR "control trial*" OR "double blind*" OR "double mask*" OR placebo*

S5 S1 AND S2 AND S3 AND S4

CINAHL (EBSCOhost)

S1 "parkinson disease" OR "parkinson's disease" OR parkinsonism

S2 pramipexol* OR rasagiline OR dexpramipexol* OR mirapex OR sifrol* OR "KNS 760704" OR KNS760704 OR "SND 919" OR "SND 919CL2x" OR SND919 OR SND919CL2x OR azilect OR "AGN 1135" OR AGN1135 OR "TVP 101" OR TVP101 OR "TVP 1022" OR TVP1022

S3 UPDRS OR "unified parkinson's disease rating scale" OR "unified parkinson disease rating scale" OR "adverse effect*" OR "adverse event*" OR "adverse reaction*" OR "adverse drug effect*" OR "adverse drug event*" OR "adverse drug reaction*" OR "side effect*" OR "injurious effect*" OR "injurious event*" OR "injurious reaction*" OR "ill effect*" OR "ill event*" OR "ill reaction*" OR harm OR harms OR harmful* OR safety OR toxic*

S4 rct OR rcts OR "randomized controlled" OR "randomised controlled" OR "random control" OR "randomized trial*" OR "randomised trial*" OR "random allocation" OR randomly OR "clinical trial*" OR "controlled trial*" OR "control trial*" OR "double blind*" OR "double mask*" OR placebo*

S5 S1 AND S2 AND S3 AND S4

Medic

Parkinson*

AND

Pramipe* rasagili* Mirapex siflor Azilect

**Supplemental Table 2.** Explanation for exclusion of study in full text screening section

|  | **Study** | **Reason for exclusion** |
| --- | --- | --- |
| 1 | Avila 2019: Rasagiline and safinamide as a dopamine-sparing therapy for Parkinson's disease | Not an RCT |
| 2 | Barone 2006: Pramipexole versus sertraline in the treatment of depression in Parkinson's disease - A national multicenter parallel-group randomized study | Duration of PD is not mentioned |
| 3 | Biglan 2007: Risk factors for somnolence, edema, and hallucinations in early Parkinson disease | Wrong outcomes |
| 4 | Blindauer 2001: Tyramine challenge to assess the safety of rasagiline monotherapy in a placebo-controlled multicenter trial for early Parkinson's disease (the TEMPO study) | Conference abstract |
| 5 | Brusa 2014: Rasagiline effect on bladder disturbances in early mild Parkinson's disease patients | Wrong outcomes |
| 6 | ChiCTR1800016949 2018: Efficacy assessment and standard treatment of rasagiline in Chinese patients with early Parkinson's disease | Full text is not available |
| 7 | Choundhry 2014: MODERATO, a randomized, double-blind, placebo-controlled study to assess the effect of rasagiline on mild cognitive impairment in patients with Parkinson's disease: an ongoing study | Conference abstract |
| 8 | CTRI/2014/05/004606 2014: Bioequivalence of Pramipexole oral prolonged release tablet in patients with idiopathic Parkinsons disease | Full text is not available |
| 9 | deMarcaida 2006: Effects of tyramine administration in Parkinson's disease patients treated with selective MAO-B inhibitor rasagiline | Mean duration of PD is more than 5 yr. |
| 10 | Eggert 2014: Influence of the nonergot dopamine agonist piribedil on vigilance in patients with Parkinson disease and excessive daytime sleepiness (PiViCog-PD): An 11-week randomized comparison trial against pramipexole and ropinirole | Duration of PD is not mentioned |
| 11 | Espay 2019: Lack of independent mood-enhancing effect for dopaminergic medications in early Parkinson's disease | Duration of PD is not mentioned |
| 12 | EUCTR2005-001416-42-AT 2005: A Multicenter, Double-Blind, Randomized Start, Placebo-Controlled, Parallel-Group Study to Assess Rasagiline as a Disease Modifying Therapy in Early Parkinson’s Disease Subjects - ADAGIO | Duplicate |
| 13 | EUCTR2005-004949-34-SE 2006: A randomized, double-blind, placebo-controlled, parallel-group clinical trial to examine the efficacy and safety of early pramipexole treatment versus delayed pramipexole treatment in patients with new onset Parkinson’s disease The main protocol includes | Duration of PD is not mentioned |
| 14 | EUCTR2006-003732-30-IT 2008: Transdermal Use of Lisuride in Early Parkinson’s Disease: a double blind, randomized, Placebo and Pramipexole controlled study to evaluate the efficacy and safety of Lisuride TTS - Tulep I | The study did not post any results |
| 15 | EUCTR2007-003353-90-FR 2007: A double-blind, double-dummy, randomized, parallel groups study to assess the Efficacy, Safety and Tolerability of switching patients with early Parkinson’s disease (PD) from Pramipexole IR to Pramipexole ER or Pramipexole IR | The study included only one intervention |
| 16 | EUCTR2007-000073-39-SK 2008: A double-blind, double-dummy, placebo-controlled, randomized, three parallel groups study comparing the Efficacy, Safety and Tolerability of Pramipexole ER versus placebo and versus Pramipexole IR administered orally over a 26-week maintenance phase in pa | Duplicate |
| 17 | EUCTR2007-004234-16-CZ 2007: Long-term safety study of open-label pramipexole extended release (ER) in patients with early Parkinson’s disease (PD) | Not an RCT |
| 18 | EUCTR2009-012419-16-DE 2009: Influence of the Non-Ergot Dopamine agonist Piribedil on vigilance and cognitive function in patients with Parkinson's disease compared to other oral Non-Ergot Dopamine agonists - PIVICOG-PD | The study did not post any results |
| 19 | EUCTR2009-013004-31-IT 2009: Early combined prolonged release ropinirole and rasagiline therapy in newly diagnosed patients with Parkinsons disease. A prospective, randomized, parallel groups, long-term follow-up study including delayed-start design of rasagiline - Early combined rop | The study did not post any results |
| 20 | EUCTR2017-001420-21-ES 2018: Clinical Efficacy, Safety and Tolerability of P2B001 in Early Parkinson's Disease | The study did not post any results |
| 21 | Gómez 2017: A multi-center comparative study of impulse control disorder in Latin American patients with Parkinson disease | Wrong outcomes |
| 22 | Goren 2010: Clinical pharmacology tyramine challenge study to determine the selectivity of the monoamine oxidase type B MAO-B) inhibitor rasagiline | Wrong patient population |
| 23 | Hattori 2018: Efficacy and safety of adjunctive rasagiline in Japanese Parkinson's disease patients with wearing-off phenomena: a phase 2/3, randomized, double-blind, placebo-controlled, multicenter study | Duplicate |
| 24 | Hattori 2018: Rasagiline monotherapy in early Parkinson's disease: a phase 3, randomized study in Japan | Duplicate |
| 25 | Hattori 2017: Efficacy and safety of rasagiline monotherapy in Japanese patients with early Parkinson's disease: A phase 3, randomized, double-blind, placebo-controlled, multicenter study | Duplicate |
| 26 | Hattori 2018: Efficacy and safety of adjunctive rasagiline in Japanese Parkinson's disease patients with wearing-off phenomena: A phase 2/3, randomized, double-blind, placebo-controlled, multicenter study | Duration of PD is more than 5 yr. |
| 27 | Hattori 2019: Long-term, open-label, phase 3 study of rasagiline in Japanese patients with early Parkinson’s disease | Not an RCT |
| 28 | Hauser 2006: Efficacy and safety of rasagiline in the treatment of Parkinson disease | Not an RCT |
| 29 | Hauser 2000: Pramipexole-induced somnolence and episodes of daytime sleep | Not an RCT |
| 30 | Hauser 2009: Long-term outcome of early versus delayed rasagiline treatment in early Parkinson's disease | Didn’t mention a stable dose of PD medication |
| 31 | Hauser 2014: Long‐term safety and sustained efficacy of extended release pramipexole in early and advanced Parkinson's disease | The study included only one intervention |
| 32 | Hauser 2013: Assess the safety and clinical benefit of rasagiline as an add-on therapy to dopamine agonist monotherapy in early Parkinson's disease (PD): the ANDANTE study | Conference abstract |
| 33 | Hauser 2014: Randomized, controlled trial of rasagiline as an add-on to dopamine agonists in Parkinson's disease | Duplicate |
| 34 | Hauser 2013: A placebo controlled, randomized, double-blind study to assess the safety and clinical benefit of rasagiline as an add-on therapy to dopamine agonist monotherapy in early Parkinson's disease (PD): The ANDANTE study | Conference abstract |
| 35 | Parkinson Study Group 2004: Pramipexole vs levodopa as initial tratment for Parkinson Disease: A 4-year randomized controlled trial | Duplicate |
| 36 | Holloway 2009: Long-term effect of initiating Pramipexole vs Levodopa in early Parkinson disease | Didn’t mention a stable dose of PD medication |
| 37 | Parkinson Study Group 2000: A randomized controlled trial comparing pramipexole with levodopa in early Parkinson's disease: Design and methods of the CALM-PD study | Duplicate |
| 38 | Hubble 1993: Pramipexole in early Parkinson's disease: a single-blind, placebo-controlled, randomized, multicenter, safety and efficacy study | Duplicate |
| 39 | Hubble 1995: Pramipexole in Patients with Early Parkinsons-Disease | Duplicate |
| 40 | Hubble 1995: Pramipexole in patients with early Parkinson's disease | Didn’t mention a stable dose of PD medication |
| 41 | Ikeda 2016: Transdermal patch of rotigotine attenuates freezing of gait in patients with Parkinson’s disease: An open-label comparative study of three non-ergot dopamine receptor agonists | Mean duration of PD is more than 5 yr. |
| 42 | Illarioshkin 2012: Rasagiline in drug-naive Russian patients with early Parkinson's disease | Full text is not available |
| 43 | Im 2019: Neuroprotective Effects of Rasagiline in Parkinson's Disease: A Regional Cerebral Blood Flow Study | Duration of PD is not mentioned |
| 44 | Isaacson 2014: Safety of rasagiline in ANDANTE: A placebo controlled, randomized, study of rasagiline as an add-on therapy to stable dose of dopamine agonists in early Parkinson's disease | Conference abstract |
| 45 | Isaacson 2013: Efficacy, safety, and tolerability of rasagiline as an add-on therapy to dopamine agonists in early Parkinson's disease: the ANDANTE study | Conference abstract |
| 46 | Jankovic 2014: Symptomatic efficacy of rasagiline monotherapy in early Parkinson's disease: post-hoc analyses from the ADAGIO trial | Wrong outcomes |
| 47 | JPRN-UMIN000006521 2011: Switch trial from Pramipexole IR (Bi-sifrol) to Pramipexole ER (Mirapex LA) in patients with Parkinson's disease | The study included only one intervention |
| 48 | Kieburtz 2011: Twice-daily, low-dose pramipexole in early Parkinson's disease: a randomized, placebo-controlled trial | Duplicate |
| 49 | Kieburtz 2009: A Randomized, controlled trial of twice daily pramipexole in early PD | Poster |
| 50 | Kieburtz 1997: Safety and efficacy of pramipexole in early Parkinson disease - A randomized dose-ranging study | Duplicate |
| 51 | Kieburtz 2011: Twice-Daily, Low-Dose Pramipexole in Early Parkinson's Disease: A Randomized, Placebo-Controlled Trial | Didn’t mention a stable dose of PD medication |
| 52 | Korchounov 2012: Combined beneficial effect of rasagiline on motor function and depression in de novo PD | The study included only one intervention |
| 53 | Kumru 2006: Increase in body weight after pramipexole treatment in Parkinson's disease | Mean duration of PD is more than 5 yr. |
| 54 | Lim 2015: Rasagiline for the symptomatic treatment of fatigue in Parkinson's disease | H&Y stage is not mentioned |
| 55 | Marek 2002: Dopamine transporter brain imaging to assess the effects of pramipexole vs levodopa on Parkinson disease progression | Wrong outcomes |
| 56 | Mizuno 2012: Efficacy and Safety of Extended-Versus Immediate-Release Pramipexole in Japanese Patients with Advanced and L-dopa-Undertreated Parkinson Disease: A Double-Blind, Randomized Trial | The study included only one intervention |
| 57 | Navan 2002: A double blind single dose cross over study of the effects of pramipexole, pergolide, and placebo on tremor and UPDRS (III) in Parkinson's disease | Didn’t mention a stable dose of PD medication |
| 58 | Navan 2003: Randomized, double-blind, 3-month parallel study of the effects of pramipexole, pergolide, and placebo on parkinsonian tremor | Didn’t mention a stable dose of PD medication |
| 59 | Navan 2003: Double-blind, single-dose, cross-over study of the effects of pramipexole, pergolide, and placebo on rest tremor and UPDRS part III in Parkinson's disease | Duplicate |
| 60 | Navan 2002: A double-blind, single-dose crossover study of the effects of pramipexole, pergolide, and placebo on tremor and UPDRS (III) in Parkinson's disease | Duplicate |
| 61 | Navana 2005: A randomly assigned double-blind cross-over study examining the relative anti-parkinsonian tremor effects of pramipexole and pergolide | Didn’t mention a stable dose of PD medication |
| 62 | NCT00144300 2005: Ophthalmologic Safety Study of Pramipexole Immediate Release (IR) Versus Ropinirole in Early Parkinson's Disease (PD) Patients | Didn’t mention a stable dose of PD medication |
| 63 | NCT00203060 2005: Effectiveness, Tolerability and Safety of Rasagiline in Early Parkinson's Disease Patients Not Treated with Levodopa | The study did not post any results |
| 64 | NCT00240409 2005: Randomized Single-blind Placebo Controlled Comparative Trial of Pramipexole and Bromocriptine in Parkinson's Disease | The study did not post any results |
| 65 | NCT00297778 2006: Pramipexole Versus Placebo in Parkinson's Disease (PD) Patients with Depressive Symptoms | Duration of PD is not mentioned |
| 66 | NCT00335166 2006: SLV 308 and Pramipexole for Treatment of Patients with Early Parkinson Disease | The study did not post any results |
| 67 | NCT00479401 2007: Efficacy, Safety, Tolerability of Pramipexol ER Versus Pramipexol IR Versus Placebo in Early PD Patients | Didn’t mention a stable dose of PD medication |
| 68 | NCT00558025 2007: Overnight Switch Trial from Pramipexole IR to Pramipexole ER in Patients with Early Parkinson Disease | The study included only one intervention |
| 69 | NCT00560508 2007: A 12-week Study of Pramipexole Extended Release (ER) in Patients with Parkinson's Disease (PD), Followed by a 52-week Long-term Treatment Period | Duration PD is not mentioned |
| 70 | NCT01048229 2010: Evaluation of the Tolerance and Acceptability of Rasagiline in the Treatment of Early-stage Parkinson's Disease | The study didn’t post any results |
| 71 | NCT01191944 2010: Pramipexole Extended Release Versus Pramipexole Immediate Release for 18 Weeks in Chinese Parkinson's Disease (PD) Patients | The study included only one intervention |
| 72 | NCT01268891 2010: Study of Azilect® (Rasagiline) in Levodopa-treated Parkinson's Disease Patients with Motor Fluctuations in Korea | The study included patients with H&Y stage III or above |
| 73 | NCT01382342 2011: The Effect of Rasagiline on Cognition in Parkinson's Disease | The study did not post any results |
| 74 | NCT01479530 2011: Azilect® (Rasagiline) in Levodopa-treated Parkinson's Patients with Motor Fluctuations in China | Duration of PD is not mentioned |
| 75 | NCT01497652 2011: A Double-Blind Placebo Controlled Trial Evaluating Rasagiline Effects on Cognition in Parkinson's Disease Patients with Mild Cognitive Impairment Receiving Dopaminergic Therapy | The study didn’t post any results |
| 76 | NCT01556165 2012: Rasagiline in Early Parkinson's Disease Patients Not Treated with Levodopa in China | Duration of PD is not mentioned |
| 77 | NCT02172573 2014: Safety and Efficacy of Pramipexole and Bromocriptine Combined with L-dopa in Parkinson's Disease | The study didn’t post any results |
| 78 | NCT02177357 2014: Pramipexole in Untreated and Levodopa-treated Parkinson's Disease Patients | The study didn’t post any results |
| 79 | O’Sullivan 1999: Oral dopamine agonists adverse drug reaction profile | Not an RCT |
| 80 | Olanow 2017: A randomized trial of a low-dose Rasagiline and Pramipexole combination (P2B001) in early Parkinson's disease | Duplicate |
| 81 | Olanow 2017: A randomized trial of a low-dose Rasagiline and Pramipexole combination (P2B001) in early Parkinson's disease | Wrong interventions |
| 82 | Parkinson 2004: A controlled, randomized, delayed-start study of rasagiline in early Parkinson disease | Didn’t mention a stable dose of PD medication |
| 83 | Playfer 2007: Ageing and Parkinson's disease | Not an RCT |
| 84 | Poewe 2015: Effects of rasagiline on the progression of nonmotor scores of the MDS-UPDRS | Wrong outcomes |
| 85 | Poewe 2015: Efficacy of rasagiline in patients with the parkinsonian variant of multiple system atrophy: A randomised, placebo-controlled trial | H&Y stage is not mentioned |
| 86 | Pogarell 2002: Pramipexole in patients with Parkinson's disease and marked drug resistant tremor: a randomised, double blind, placebo controlled multicenter study | Mean duration of PD is more than 5 yr. |
| 87 | Rabey 2000: Rasagiline mesylate, a new Mao-B inhibitor for the treatment of Parkinson's disease: A double-blind study as adjunctive therapy to levodopa | Mean duration of PD is more than 5 yr. |
| 88 | Rascol 2005: Rasagiline as an adjunct to levodopa in patients with Parkinson's disease and motor fluctuations (LARGO, Lasting effect in Adjunct therapy with Rasagiline Given Once daily, study): a randomised, double-blind, parallel-group trial | Mean duration of PD is more than 5 yr. |
| 89 | Rascol 2010: Efficacy, safety, and tolerability of overnight switching from immediate- to once daily extended-release pramipexole in early Parkinson's disease | No comparison intervention |
| 90 | Schapira 2010: Rationale for delayed-start study of pramipexole in Parkinson's disease: The PROUD study | The study did not post any results |
| 91 | Schwid 2005: A randomized placebo-controlled trial of rasagiline in levodopa-treated patients with Parkinson disease and motor fluctuations - The PRESTO study | Mean duration of PD is more than 5 yr. |
| 92 | Seiple 2016: Ophthalmologic Baseline Characteristics and 2-Year Ophthalmologic Safety Profile of Pramipexole IR Compared with Ropinirole IR in Patients with Early Parkinson’s Disease | Didn’t mention a stable dose of PD medication |
| 93 | Shannon 1997: Efficacy of pramipexole, a novel dopamine agonist, as monotherapy in mild to moderate Parkinson's disease. The Pramipexole Study Group | Duplicate |
| 94 | Shepherd 2018: Feasibility of a randomized single-blind crossover trial to assess the effects of the second-generation slow-release dopamine agonists pramipexole and ropinirole on cued recall memory in idiopathic mild or moderate Parkinson's disease without cognitive im | Duration of PD is not mentioned |
| 95 | Siderowf 2004: A controlled, randomized, delayed-start study of rasagiline in early Parkinson disease | The study included only one intervention |
| 96 | Siderowf 2002: A controlled trial of rasagiline in early Parkinson disease - The TEMPO study | Duplicate |
| 97 | Siderowf 2002: A Controlled Trial of Rasagiline in Early Parkinson Disease | Didn’t mention a stable dose of PD medication |
| 98 | Silver 2013: Efficacy, safety, and tolerability of rasagiline as add-on to suboptimal dopamine agonist monotherapy in Parkinson's disease (PD): the ANDANTE study | Conference abstract |
| 99 | Smith 2014: Combined rasagiline and antidepressant use in Parkinson's disease in the ADAGIO study: effects on non-motor symptoms and tolerability | Poster |
| 100 | Smith 2015: Combined rasagiline and antidepressant use in Parkinson disease in the ADAGIO study: Effects on nonmotor symptoms and tolerability | Wrong outcomes |
| 101 | Stocchi 2011: Effect of rasagiline as adjunct therapy to levodopa on severity of OFF in Parkinson’s disease | Mean duration of PD is more than 5 yr. |
| 102 | Stocchi 2014: Phase-3 clinical trial of the adenosine 2a antagonist preladenant, given as monotherapy, in patients with Parkinson’s disease | Stable dose of other PD medication was less than 4 weeks |
| 103 | Stocchi 2017: Randomized trial of preladenant, given as monotherapy, in patients with early Parkinson disease | Didn’t mention a stable dose of PD medication |
| 104 | Tanner 2007: Pramipexole in levodopa-treated Parkinson disease patients of African, Asian, and Hispanic heritage | Mean duration of PD is more than 5 yr. |
| 105 | Tanner 2007: Pramipexole in levodopa-treated Parkinson disease patients of African, Asian, and Hispanic heritage | Duplicate |
| 106 | Utsumi 2013: Evaluation of the efficacy of pramipexole for treating levodopa-induced dyskinesia in patients with Parkinson's disease | Mean duration of PD is more than 5 yr. |
| 107 | Vanacore 2010: What is the clinical significance of the findings from the delayed-start trial of rasagiline in Parkinson’s disease? | Not an RCT |
| 108 | Viallet 2011: A comparative study on safety and tolerability of rasagiline versus pramipexole in the treatment of early Parkinson’s disease: the actor study | Poster |
| 109 | Viallet 2012: A comparative study on safety and tolerability of rasagiline versus pramipexole in early Parkinson's disease (PD): the ACTOR study | Poster |
| 110 | Wang 2014: The efficacy and safety of pramipexole ER versus IR in Chinese patients with Parkinson's disease: A randomized, double-blind, double-dummy, parallel-group study | The study included only one intervention |
| 111 | Weintraub 2013: A randomized, double blind, placebo-controlled study to assess the effect of rasagiline on mild cognitive impairment in patients with Parkinson's disease: the MODERATO study | Poster |
| 112 | Weintraub 2016: Efficacy of rasagiline in PD patients with mild cognitive impairment | Conference abstract |
| 113 | Weintraub 2015: Efficacy of rasagiline in patients with Parkinson's disease and mild cognitive impairment: results from moderato, a 24-week randomized, double-blind, placebo-controlled trial | Duration of PD is not mentioned |
| 114 | Weintraub 2015: Moderato, a randomized, double-blind, placebo-controlled study to assess the effect of rasagiline on mild cognitive impairment in patients with Parkinson’s disease: an ongoing study | Conference abstract |
| 115 | Weintraub 2016: Rasagiline for mild cognitive impairment in Parkinson's disease: A placebo-controlled trial | Duration of PD is not mentioned |
| 116 | Wen 2006: Efficacy of pramipexole in the treatment of Parkinson's disease: a multi-center, randomized, double-blind, bromocriptine-control trial | Full text is not available |
| 117 | Yun 2014: Efficacy and safety of Rasagiline in levodopa-treated Korean Parkinson's disease patients with motor fluctuations: Randomized, placebo-controlled, double-blind study | Poster |
| 118 | Zhang 2013: Efficacy and safety of Rasagiline in levodopa-treated Korean Parkinson's disease patients with motor fluctuations: Randomized, placebo-controlled, double-blind study | Mean duration of PD is more than 5 yr. |
| 119 | Zhang 2010: Efficacy observation of Pramipexole add-on therapy in Parkinson's disease | Full text is not available |
| 120 | Zhang 2018: Adjunct rasagiline to treat Parkinson's disease with motor fluctuations: a randomized, double-blind study in China | Wrong patient population |
| 121 | Zhang 2018: Efficacy and safety of rasagiline in Chinese patients with early Parkinson's disease: a randomized, double-blind, parallel, placebo-controlled, fixed-dose study | Didn’t mention a stable dose of PD medication |
| 122 | No author 1997: Safety and efficacy of pramipexole in early Parkinson disease. A randomized dose-ranging study. Parkinson Study Group | Duplicate |
| 123 | No author 2000. Pramipexole vs levodopa as initial treatment for Parkinson disease: A randomized controlled trial. Parkinson Study Group | Duplicate |
| 124 | No author 2000: Pramipexole vs levodopa as initial treatment for Parkinson disease: A randomized control trial | Duplicate |
| 125 | No author 2001: Pramipexole in the treatment of early Parkinson's disease | Not an RCT |
| 126 | No author 2007: Pramipexole in levodopa-treated Parkinson disease patients of African, Asian, and Hispanic heritage | Mean duration of PD is more than 5 yr. |
| 127 | No author 2015: Pioglitazone in early Parkinson's disease: a phase 2, multicenter, double-blind, randomised trial | Wrong intervention |
| 128 | No author 2001: Parkinson's disease - Rasagiline | Not an RCT |
| 129 | No author 2002: Dopamine transporter brain imaging to assess the effects of pramipexole vs levodopa on Parkinson disease progression | Duplicate |

**Supplemental Table 3. Adverse events reported in patients receiving rasagiline (significant differences are highlighted using bold text)**

|  | Barone *et al.* 2015 | Hanagasi *et al.* 2011 | Hattori *et al.* 2019 | Hauser *et al.* 2014 | Olanow *et al.* 2009 ^a^ | Schrempf *et al.* 2018 | Stern *et al.* 2004 | Viallet *et al.* 2013 |
| --- | --- | --- | --- | --- | --- | --- | --- | --- |
| Dose (mg/day)  Study duration (wk.)  Population (n) | 1  12 wk.  65 (PBO)  58 (RA) | 1  12 wk.  25 (PBO)  23 (RA) | 1  26 wk.  126 (PBO)  118 (RA) | 1  18 wk.  164 (PBO)  162 (RA) | 1, 2  72 wk. (reported ≤ 36 wk.)  595 (PBO)  288 (RA 1 mg)  293 (RA 2 mg) | 1  8 wk.  10 (PBO)  20 (RA) | 1, 2, 4  10 wk.  13 (PBO)  15 (RA 1 mg)  14 (RA 2 mg)  14 (RA 4 mg) | 1,5 (PPX), 1 (RA)  15 wk.  56 (PPX)  53 (RA) |
| **Adverse events** | **Incidence (%)** | | | | | | | |
| Any | 26.2 (PBO)  25.9 (RA) | 5.5 (PBO + RA) | 52.4 (PBO)  62.4 (RA) | 61.0 (PBO)  64.2 (RA) | - | 40.0 (PBO)  60.0 (RA) | 61.5 (PBO)  65.1 (RA) | 76.8 (PPX)  67.9 (RA) |
| Serious | 1.5 (PBO)  3.4 (RA) | - | 6.3 (PBO)  3.4 (RA) | 3.0 (PBO)  4.9 (RA) | - | 0 (PBO)  5.0 (RA) | 0 (PBO)  0 (RA) | 1.8 (PPX)  1.9 (RA) |
| Anxiety | - | - | - | - | 5.7 (PBO)  3.5 (RA 1)  3.1 (RA 2) | - | - | 7.1 (PPX)  7.5 (RA) ^b^ |
| Back pain | - | 1.8 (PBO + RA) ^c^ | 4.0 (PBO)  2.6 (RA) | - | 5.4 (PBO)  4.9 (RA 1)  5.1 (RA 2) | - | - | - |
| Common cold | - | - | - | - | - | 0 (PBO)  10.0 (RA) | - | - |
| Confusion | - | - | 4.8 (PBO)  3.4 (RA) | - | - | - | - | - |
| Constipation | - | - | - | - | - | - | - | 7.1 (PPX)  3.8 (RA) |
| Death | - | - | 0.8 (PBO) ^d^  0 (RA) | - | - | - | - | - |
| Depression | - | - | - | - | 6.1 (PBO)  3.5 (RA 1)  3.4 (RA 2) | - | - | - |
| Diarrhea | - | - | - | - | - | - | 23 (PBO)  12 (RA) | - |
| Dizziness | - | - | - | 6.1 (PBO)  7.4 (RA) | - | 0 (PBO)  15.0 (RA) | 15 (PBO)  23 (RA) | 8.9 (PPX)  5.7 (RA) |
| Dyskinesia | 0 (PBO)  ≤ 6.9 (RA) | - | - | - | - | - | - | - |
| Eczema | - | - | 1.6 (PBO)  4.3 (RA) | - | - | - | - | - |
| Edema | - | - | - | - | - | - | 0 (PBO)  2 (RA) | - |
| Fall | - | - | 5.6 (PBO)  5.1 (RA) | 1.2 (PBO)  5.6 (RA) | - | - | - | - |
| Fatigue | - | - | - | - | 2.9 (PBO)  5.9 (RA 1)  3.4 (RA 2) | - | - | - |
| Gastralgia | - | - | - | - | - | - | - | 8.9 (PPX)  7.5 (RA) |
| Hallucination | - | - | - | - | 0.2 (PBO)  0 (RA 1)  0.3 (RA 2) | 0 (PBO) ^e^  15.0 (RA) | 0 (PBO)  2 (RA) | 5.4 (PPX)  0 (RA) |
| Headache | - | 3.6 (PBO + RA) | 0.8 (PBO)  3.4 (RA) | 4.3 (PBO)  6.2 (RA) | 6.2 (PBO)  4,9 (RA 1)  5.1 (RA 2) | 0 (PBO)  10.0 (RA) | 31 (PBO)  26 (RA) | 8.9 (PPX)  5.7 (RA) |
| Hypersexuality | - | - | - | - | 0 (PBO)  0 (RA 1)  0.3 (RA 2) | - | - | - |
| Hypertension | - | - | - | - | 3.9 (PBO)  1.7 (RA 1)  2.4 (RA 2) | - | - | - |
| Infection | - | - | - | - | - | - | 31 (PBO)  12 (RA) | 8.9 (PPX) ^f^  7.5 (RA) |
| Insomnia | - | - | - | - | - | - | 0 (PBO)  12 (RA) | - |
| Itching | - | - | - | - | - | - | - | 0 (PPX)  5.7 (RA) |
| Malaise, syncope | - | - | - | - | - | - | - | 10.7 (PPX)  7.5 (RA) |
| Melanocytic nevus | 0 (PBO)  0.8 (RA) | - | - | - | - | - | - | - |
| Mood swings | - | - | - | - | - | - | - | 7.1 (PPX)  9.4 (RA) |
| Muscle cramps | - | - | - | - | - | - | - | 3.6 (PPX)  9.4 (RA) |
| Myalgia | - | - | - | - | - | 10.0 (PBO)  5.0 (RA) | - | - |
| Nasopharyngitis | - | - | 15.1 (PBO)  15.4 (RA) | - | 5.4 (PBO)  4.2 (RA 1)  3.8 (RA 2) | - | - | - |
| Nausea | 0 (PBO)  ≤ 6.9 (RA) | - | - | 4.3 (PBO)  6.2 (RA) | 3.9 (PBO) ^g^  4.2 (RA 1)  2.7 (RA 2) | 10.0 (PBO)  5.0 (RA) | 8 (PBO)  7 (RA) | **28.6 (PPX) (P=0.011)** ^g^  9.4 (RA) |
| Orthostatic hypotension | - | - | - | - | 0.8 (PBO)  0.7 (RA 1)  0.3 (RA 2) | - | - | 5.4 (PPX)  1.9 (RA) |
| Sleep disorders, daytime sleepiness | - | - | - | - | - | - | - | **35.7 (PPX) (P=0.027)**  17.0 (RA) |
| Somnolence | - | - | - | 6.7 (PBO)  6.8 (RA) | 1.5 (PBO)  0.7 (RA 1)  1.4 (RA 2) | - | 0 (PBO)  5 (RA) | - |
| Tingling | - | - | - | - | - | - | - | 3.6 (PPX)  7.5 (RA) |
| Trunk flexion | 0 (PBO)  ≤ 6.9 (RA) | - | - | - | - | - | - | - |
| Pain | - | - | - | - | - | - | 15 (PBO)  30 (RA) | 21.4 (PPX) ^h^  13.2 (RA) |
| Paresthesia | - | - | - | - | - | - | 0 (PBO)  12 (RA) | - |
| Peripheral edema | - | - | - | 4.3 (PBO)  7.4 (RA) | - | - | - | - |
| Radius fracture | 0 (PBO)  0.8 (RA) | - | - | - | - | - | - | - |
| Rash | - | - | - | - | - | - | - | 0 (PPX)  **9.4 (RA) (P=0.025)** |
| Tremor | - | - | - | 6.1 (PBO)  4.3 (RA) | - | - | - | - |
| Vertigo | 0 (PBO)  ≤ 6.9 (RA) | - | - | - | - | - | - | - |
| Weakness | - | - | - | - | - | - | - | 12.5 (PPX)  11.3 (RA) |
| Weight gain | - | - | - | - | - | - | - | 7.1 (PPX)  3.8 (RA) |
| Weight loss | - | - | - | - | - | - | - | 0 (PPX)  5.7 (RA) |
| Withdrawals due AEs | 0 (PBO)  6.9 (RA) | 0 (PBO)  0 (RA) | 6.3 (PBO)  2.6(RA) | 4.3 (PBO)  8.0 (RA) | 2.9 (PBO)  3.1 (RA 1 mg)  3.8 (RA 2 mg) | 10.0 (PBO)  5.0 (RA) | 0 (PBO)  6.7 (RA 1 mg)  0 (RA 2 mg)  0 (RA 4 mg) | 14.3 (PPX)  5.7 (RA) |
| Comment | Four RA patients developed dyskinesia, vertigo, flexion, or nausea. | AE data not segregated by treatment group. | Data missing for 1 patient in RA group | Data missing for one patient in each group | - | - | AE data pooled across all RA dose groups | - |

Dose reported as mean ± standard deviation

Abbreviations: AE = adverse event, NR = not reported, TEAE = treatment emerging adverse effect, PBO = placebo, PPX = pramipexole, RA = rasagiline, WK = weeks

^a^ Adverse events that appeared before week 36 and active treatment phase

^b^ Anxiety, irritability, emotionality

^c^ Low back pain

^d^ Suicide, this was judged to be unlikely to be related to the drug treatment

^e^ Pseudohallucinations / delusion

^f^ Respiratory infection

^g^ Nausea, vomiting

^h^ Joint pain, joint disease

**Supplemental Table 4. Adverse events reported in patients receiving pramipexole (significant differences are highlighted using bold text). See Supplemental Table 3 for data for Vialet et al., (2013)**

|  |  |  |  |  |  |  |  |  |  |
| --- | --- | --- | --- | --- | --- | --- | --- | --- | --- |
|  | Barone *et al.* 2010 | Hauser *et al.* 2010 | PSG *et al.* 2000, PSG *et al.* 2004 | Kieburtz *et al.* 1997 | Poewe *et al.* 2011 | Schapira *et al.* 2013 | Shannon *et al.* 1997 | Thomas *et al.* 2006 | Wong *et al.* 2003 |
| Dose (mg/day)  Study duration (wk.)  Population (n) | 0.375-4.5  2.18 ± 0.83  12 wk.  152 (PBO)  144 (PPX) | 3.05 ± 1.37 (ER)  3.03 ± 1.39 (IR)  18 wk.  50 (PBO)  106 (ER)  103 (IR) | 427 ± 112 (LD) ^a^  2.78 ± 1.1 (PPX)  23.5-48 mo.  150 (LD)  151 (PPX) | 1.5, 3.0, 4.5, 6.0  10 wk.  51 (PBO)  54 (PPX 1.5)  50 (PPX 3.0)  54 (PPX 4.5)  55 (PPX 6.0)  213 (PPX) | 0.375-4.5  2.9 ± 1.4 ER and IR  33 wk.  103 (PBO)  223 (ER)  213 (IR) | 1.5  6 to 9 mo. ^b^  274 (PBO)  261 (PPX) | 0.375-4.5  mean 3.8  31 wk.  171 (PBO) 164 (PPX) | 15 (RR), 2.1 (PPX)  24 mo.  30 (RR)  30 (PPX) | 0.375-4.5  15 wk.  77 (PBO)  73 (PPX) |
| **Adverse events** | **Incidence (%)** | | | | | | | | |
| Any | 66.4 (PBO)  72.9 (PPX) | 70 (PBO)  76.4 (ER)  76.8 (IR) | - | 78.4 (PBO)  79.6 (PPX 1.5)  84.0 (PPX 3.0)  87.0 (PPX (4.5)  89.1 (PPX 6.0)  85.0 (PPX) | 77.7 (PBO)  84.8 (ER)  80.8 (IR) | 71.5 (PBO)  74.3 (PPX) | - | - | 71.4 (PBO)  86.3 (PPX) |
| Severe | 3.9 (PBO)  8.3 (PPX) | 2.0 (PBO)  3.8 (ER)  5.8 (IR) | - | 37.3 (PBO) ^c^  44.4 (PPX 1.5)  36.0 (PPX 3.0)  42.6 (PPX 4.5)  67.3 (PPX 6.0)  47.9 (PPX) | 3.9 (PBO)  5.4 (ER)  5.2 (IR) | 8.4 (PBO)  13.0 (PPX) | - | - | - |
| Serious | 3.9 (PBO)  4.2 (PPX) | 2.0 (PBO)  4.7 (ER)  2.9 (IR) | - | - | 3.9 (PBO)  7.2 (ER)  5.2 (IR) | 6.6 (PBO)  6.5 (PPX) | - | - | 3.9 (PBO)  2.7 (PPX) |
| Drug related AE | - | - | - | - | 38.8 (PBO)  63.2 (ER)  62.9 (IR) | - | - | - | - |
| Abnormal dreams |  |  | 12.7 (LD)  13.9 (PPX) |  |  |  |  |  |  |
| Anxiety | - | - | 6.7 (LD)  11.3 (PPX) | - | - | - | - | - | - |
| Asthenia | - | - | - | - | - | - | 2.3 (PBO)  6.1 (PPX) | - | - |
| Back pain | - | - | - | - | - | 4.7 (PBO)  5.4 (PPX) | - | - | - |
| Cellulitis | - | - | 0 (LD)  **4.6 (PPX) (P=0.01)** | - | - | - | - | - | - |
| Compulsive behavior ^d^ |  |  |  |  | 1.9 (PBO)  2.2 (ER)  1.4 (IR) |  |  |  |  |
| Confusion | - | - | - | 0 (PBO)  5.6 (PPX 1.5)  4.0 (PPX 3.0)  1.9 (PPX 4.5)  5.5 (PPX 6.0)  4.2 (PPX) | - | - | - | - | - |
| Constipation | - | 0 (PBO)  12.3 (ER)  15.5 (IR) | 20.5 (PPX)  12.7 (LD) | 5.9 (PBO)  7.4 (PPX 1.5)  12.0 (PPX 3.0)  5.6 (PPX 4.5)  18.2 (PPX 6.0)  10.8 (PPX) | 1.9 (PBO)  14.3 (ER)  11.7 (IR) | 7.3 (PBO)  6.1 (PPX) | 6.4 (PBO)  **17.7 (PPX) (P=0.0021)** | - | 5.2 (PBO)  20.6 (PPX) |
| Depression | - | - | 15.2 (PPX)  13.3 (LD) | - | - | 4.4 (PBO)  5.0 (PPX) | - | - | - |
| Diarrhea |  |  |  |  |  | 5.5 (PBO)  3.1 (PPX) |  |  |  |
| Dizziness | 5.9 (PBO)  8.3 (PPX) | - | 25.8 (PPX)  24.0 (LD) | 19.6 (PBO)  18.5 (PPX 1.5)  20.0 (PPX 3.0)  16.7 (PPX 4.5)  18.2 (PPX 6.0)  18.3 (PPX) | 6.8 (PBO)  11.7 (ER)  11.7 (IR) | 8.8 (PBO)  11.1 (PPX) | - | - | 31.2 (PBO)  27.4 (PPX) |
| Dry mouth | - | - | - | - | 1.0 (PBO)  5.4 (ER)  3.8 (IR) | - | - | - | 5.2 (PBO)  12.3 (PPX) |
| Dyskinesia | 2.0 (PBO)  6.9 (PPX) |  | **54.0 (LD) (P<0.001)**  24.5 (PPX) |  |  |  |  |  | 5.2 (PBO)  12.3 (PPX) |
| Edema | - | - | 14.7 (LD)  **42.4 (PPX) (P<0.001) ^e^** | - | - | - | - | - | - |
| Fatigue | 3.3 (PBO)  4.2 (PPX) | 2.0 (PBO)  6.6 (ER)  6.8 (IR) | - | 9.8 (PBO)  7.4 (PPX 1.5)  4.0 (PPX 3.0)  3.7 (PPX 4.5)  10.9 (PPX 6.0)  6.6 (PPX) | - | 7.7 (PBO)  10.0 (PPX) | 8.8 (PBO)  14.6 (PPX) | - | - |
| Freezing | - | - | 25.3 (LD)  **37.1 (PPX) (P=0.01)** | - | - | - | - | - | - |
| Hallucinations | - | - | 8.0 (LD)  14.6 (PPX) | 0 (PBO)  7.4 (PPX 1.5)  8.0 (PPX 3.0)  1.9 (PPX 4.5)  9.1 (PPX 6.0)  6.6 (PPX) | - | 1.1 (PBO)  5.0 (PPX) | 2.3 (PBO)  11.5 (PPX) ^f^ | - | 1.3 (PBO)  12.3 (PPX) |
| Hernia |  |  | **8.0 (LD) (P=0.002)**  0.7 (PPX) |  |  |  |  |  |  |
| Headaches | 3.9 (PBO)  4.9 (PPX) | - | 15.3 (LD)  20.5 (PPX) | 9.8 (PBO)  9.2 (PPX 1.5)  14.0 (PPX 3.0)  14.8 (PPX 4.5)  7.3 (PPX 6.0)  11.3 (PPX) | - | 8.4 (PBO)  6.5 (PPX) | - | - | - |
| Insomnia | 2.0 (PBO)  3.5 (PPX) | - | 22.0 (LD)  25.8 (PPX) | 7.8 (PBO)  3.7 (PPX 1.5)  4.0 (PPX 3.0)  13.0 (PPX 4.5)  9.1 (PPX 6.0)  7.5 (PPX) | - | 2.9 (PBO)  6.5 (PPX) | 12.9 (PBO)  **25.6 (PPX) (P=0.0034)** | - | - |
| Musculoskeletal pain | - | - | - | 19.6 (PBO)  14.8 (PPX 1.5)  12.0 (PPX 3.0)  5.6 (PPX 4.5)  7.3 (PPX 6.0)  9.8 (PPX) | - | - | - | - | - |
| Nasopharyngitis | - | - | - | - | - | 5.5 (PBO)  6.1 (PPX) | - | - | - |
| Nausea | 13.1 (PBO)  13.9 (PPX) | 4.0 (PBO)  20.8 (ER)  21.4 (IR) | 36.7 (LD)  36.4 (PPX) | 9.8 (PBO)  16.7 (PPX 1.5)  18.0 (PPX 3.0)  22.2 (PPX 4.5)  21.8 (PPX 6.0)  19.7 (PPX) | 8.7 (PBO)  21.5 (ER)  23.9 (IR) | 7.7 (PBO)  20.7 (PPX) | 20.5 (PBO)  **39.0 (PPX) (P=0.0002)** | - | 1.3 (PBO)  15.1 (PPX) |
| Off-period dystonia | - | - | 46.0 (LD)  35.1 (PPX) | - | - | - | - | - | - |
| On-off fluctuations | - | - | 8.0 (LD)  6.6 (PPX) | - | - | - | - | - | - |
| Peripheral edema | - | - | 6.0 (LD)  **22.5 (PPX) (P<0.001)** | - | - | 1.5 (PBO)  6.5 (PPX) | 3.5 (PBO)  7.9 (PPX) | - | - |
| Postural hypotension |  |  | 10.0 (LD)  6.0 (PPX) |  |  |  | 5.6 (PBO) ^g^  9.8 (PPX) |  |  |
| Somnolence | 7.9 (PBO)  8.3 (PPX) | 14.0 (PBO)  32.1 (ER)  33.0 (IR) | 21.3 (LD)  **36.4 (PPX) (P=0.005)** | 13.7 (PBO)  16.7 (PPX 1.5)  30.0 (PPX 3.0)  31.5 (PPX 4.5)  30.9 (PPX 6.0)  27.2 (PPX) | 14.6 (PBO)  36.3 (ER)  32.9 (IR) | 3.3 (PBO)  10.7 (PPX) | 8.8 (PBO)  **18.3 (PPX) (P=0.015)** | 3.3 (PPX + RR) | 7.8 (PBO)  12.3 (PPX) |
| Tremor | - | - | - | - | - | - | - | - | 11.7 (PBO)  1.4 (PPX) |
| Urinary frequency | - | - | **10.7 (LD) (P=0.01)**  3.3 (PPX) | - | - | - | - | - | - |
| Vertigo | 2.0 (PBO)  4.2 (PPX) | - | - | - | - | - | - | - | - |
| Vomiting | - | - | - | - | - | - | 4.7 (PBO)  1.8 (PPX) | - | - |
| Wearing off | - | - | **62.7 (LD) (P=0.02)**  47.0 (PPX) | - | - | - | - | 32.0 (PPX)  37.0(RR) | - |
| Withdrawals due AEs | 10.5 (PBO)  6.9 (PPX) | 4.0 (PBO)  10.4 (ER)  7.8 (IR) | 22.7 (LD)  32.5 (PPX) | 0 (PBO)  9.9 (PPX) | 3.9 (PBO)  10.8 (ER)  9.4 (IR) | 9.5 (PBO)  9.6 (PPX) | 14.0 (PBO)  13.4 (PPX) | 10.0 (PPX)  16.7 (RR) | 3.9 (PBO)  8.2 (PPX) |
| Comments | Treatment related AEs | - | - | There was a higher incidence of moderate and severe adverse experiences in the 6.0 mg/d group (nominal P=.002). | - | Values reported at 9 mo. which was at the end of placebo treatment | - | 10.0 (PPX + RR) withdrew due to gastrointestinal AEs | - |

Dose reported as mean ± standard deviation

Abbreviations: AE = adverse event, ER = extended release, IR = immediate release, LD = levodopa, MO = months, PBO = placebo, PPX = pramipexole, PSG: Parkinson Study Group, RR = ropinirole, WK = weeks

^a^ 59.3% in LD group required supplemental open-label LD. Mean (± SD) dosage 274 ± 442. 72.1% in PPX group required open-label LD. Mean (± SD) dosage 434 ± 498 mg/day.

^b^ Patients were assigned in the pramipexole group at 9 months or as early as 6 months, if the patients expressed inability to tolerate PD symptoms.

^c^ Moderate and severe intensity

^d^ Pathologic gambling, compulsive buying, compulsive checking and washing, compulsive sexual behavior, loss of self-inhibition and increased alcohol consumption

^e^ Peripheral edema, localized edema, generalized edema, facial edema, tongue edema, periorbital edema, and lymphedema

^f^ Visual hallucinations 2.3 (PBO), 9.7 (PPX) (P=0.0048). Auditory hallucinations 0 (PBO), 1.8 (PPX) (P=0.116).

^g^ Orthostatic blood pressure change > 20 mmHg systolic
